# Hobby engagement, disability transitions, and life expectancy: a multinational longitudinal study

**DOI:** 10.7189/jogh.16.04127

**Published:** 2026-05-08

**Authors:** Qian Liu, Fan Yang

**Affiliations:** 1 School of Public Health, Fudan University, Shanghai, China; 2 National Health Commission Key Lab of Health Technology Assessment, Fudan University, Shanghai, China

## Abstract

**Background:**

Hobby engagement has been identified as a potentially modifiable determinant of healthy ageing, associated with reduced mortality and better cognitive outcomes. However, its role in functional disability transitions and life expectancy across diverse cultural contexts remains insufficiently characterised. In this study, we aimed to examine associations between hobby engagement and functional disability transitions among middle-aged and older adults across Mexico, UK, China, USA, and Europe, and to further quantify the life expectancy implications of hobby engagement across diverse cultural contexts.

**Methods:**

We conducted a prospective cohort study using harmonised data from five major longitudinal ageing surveys, encompassing 127 650 participants aged >50 years from 24 countries. Hobby engagement was assessed as binary participation in activities such as volunteering, clubs, reading, or games. Functional disability states were defined as no disability, mild disability, severe disability, and death. Using continuous-time multi-state Markov models, we analysed transitions between states, with life expectancy difference measures calculated at five-year intervals from ages 50–90.

**Results:**

Hobby engagement prevalence varied substantially across regions (25.2–83.3%). Hobby participation was associated with a lower risk of transitioning from no disability to mild disability, a higher likelihood of recovery from severe disability to functional independence, and a lower mortality risk from severe disability across the four countries and Europe. These associations translated into differences in life expectancy. At the age of 50 years, functional life expectancy differences ranged from 4.73–5.17 years, and total life expectancy differences ranged from 2.46–4.64 years across the four countries and Europe, with associations between hobby engagement and life expectancy persisting through age 90 (except in China). Life expectancy differences varied by gender, chronic disease status, and geographic location in some countries.

**Conclusions:**

Hobby engagement is consistently associated with favourable functional disability transitions and longer life expectancy across diverse cultural contexts, with findings remaining stable in sensitivity analyses, supporting its consideration in healthy ageing strategies.

Functional disability transitions among adults aged >50 years represent a critical global health challenge. Rather than static conditions, functional disabilities are characterised by dynamic transitions between four distinct states: no disability, mild disability, severe disability, and death [1,2]. Recent longitudinal evidence demonstrates that these transitions allow bidirectional movement between all states except death. Analysis of Shanghai Elderly Care data revealed that adults with mild activities of daily living (ADL) disability were more likely to improve (transition intensity = 0.4731) than to deteriorate (transition intensity = 0.2226) [2]. These findings highlight critical opportunities for intervention at different transition points to prevent the onset of disability, halt deterioration, and facilitate recovery toward functional independence.

Hobby engagement has emerged as a potentially modifiable determinant of functional outcomes in ageing populations. Hobbies are defined as enjoyable leisure activities involving active participation during free time, including arts, crafts, reading, games, sports, gardening, volunteering, and social club participation [3,4]. Distinguished from passive leisure activities, hobby engagement incorporates cognitive stimulation, creativity, physical activity, and social interaction components that may influence disability transitions through multiple pathways. Compelling evidence demonstrates substantial protective effects across health domains. A longitudinal study of 22 377 individuals aged 40–69 years found hobby engagement inversely associated with incident disabling dementia, with multivariable-adjusted hazard ratios (HR) of 0.82 (95% confidence interval (CI) = 0.75, 0.89) [4]. Furthermore, international analysis of 79 464 adults aged >50 years across 19 countries revealed a 29% reduction in all-cause mortality risk associated with hobby engagement (pooled HR = 0.71; 95% CI = 0.67, 0.75) [5].

However, evidence specifically examining hobby engagement and disability transitions remains limited. Most studies have focused on leisure activities broadly rather than hobby engagement specifically. A prospective study of 3241 elderly individuals demonstrated that leisure-time physical activities reduced the risk of decline in instrumental activities of daily living (IADL) (adjusted risk ratio = 0.73; 95% CI = 0.60, 0.89), with cultural leisure activities showing similar protective effects [6]. Longitudinal analysis using Chinese Longitudinal Healthy Longevity Survey data found that leisure activities had significant inhibitory effects on ADL disorder, IADL disorder, and cognitive impairment among older adults [7]. Nevertheless, these studies have not examined the dynamic transitions between different disability states that characterise the ageing process.

Disability transitions have important implications for life expectancy outcomes. Multiple factors influence life expectancy and years lived with disability, including disability incidence and recovery rates, as well as mortality risk associated with disability [8,9]. Research demonstrates substantial differences in life expectancy based on disability status. Mexican data showed that at the age of 60 years, individuals without ADL limitations live approximately 11 years longer than those with initial disabilities [10], while USA data revealed similar patterns with 65-year-old adults without limitations having 2.8 years longer life expectancy and 5.9 years more disability-free life expectancy [11]. These disability-related life expectancy patterns reflect a global phenomenon with substantial international variations. International research across the Asia-Pacific regions, analysing 778 507 individuals aged >45 years across six countries, demonstrates significant variations in functional limitations [12]. Similarly, hobby engagement patterns demonstrate substantial international variation, with prevalence ranging from 23.3% in China to over 97% in Northern European countries [5]. Whether hobby engagement influences life expectancy outcomes through its effects on disability transitions remains unexplored.

To address these critical knowledge gaps, we conducted a comprehensive multinational analysis of longitudinal associations between hobby engagement and transitions across ADL and IADL disability states among adults aged >50 years. Disability transition risks were the primary analytical focus, and life expectancy estimation was an extended analysis to characterise the cumulative patterns associated with these transitions. Using data from the family of health and retirement studies, including the China Health and Retirement Longitudinal Study (CHARLS), Health and Retirement Study (HRS), English Longitudinal Study of Ageing (ELSA), Mexican Health and Aging Study (MHAS), and Survey of Health, Ageing and Retirement in Europe (SHARE), provides unprecedented opportunities for this study because these harmonised longitudinal studies employed similar methodologies while representing diverse cultural contexts [2,6].

In this study, we adhered to GRABDROP guidelines (Table S1 in the **Online Supplementary Document**) [13].

## METHODS

### Study design and participants

We examined data from CHARLS waves 1–4 (years 2011–2018), MHAS waves 3–5 (years 2012–2018), SHARE waves 4–8 (years 010–2019), ELSA waves 1–6 (years 2002–2012), and HRS waves 9–15 (years 2008–2020), encompassing 127 650 participants aged >50 years across 24 countries (Methods S1 in the **Online Supplementary Document**). Wave selection was determined by concurrent availability of hobby engagement measures and functional disability assessments across all surveys. For ELSA, we restricted analyses to waves 1–6 when linked mortality information was available, as death serves as an absorbing state in our multi-state models. These wave selections align with prior research examining hobby engagement using these harmonised cohorts [5]. We conducted and reported this study in accordance with the STROBE reporting guidelines [14].

### Sample selection

The proportions of participants aged ≥50 years in each original cohort sample were: 66.80% in CHARLS, 92.21% in MHAS, 93.81% in ELSA, 94.26% in HRS, and 97.37% in SHARE. We included participants aged ≥50 years at baseline. Exclusion criteria were: missing disability state information at any assessment wave, missing hobby engagement or any covariate at a given wave (complete-case analysis, no imputation was applied), and availability of only single-wave disability data. This approach is consistent with established methodology in multi-state modelling [10,15,16]. Following these inclusion and exclusion criteria, the analytical sample comprised participants who provided complete time-varying information and sufficient longitudinal observations for disability transition modelling (Figure S1–5 in the **Online Supplementary Document**).

### Assessment of hobby engagement

Hobby engagement served as the primary independent variable, measured using harmonised questions across the five cohort studies. The measurement approaches and response categories varied slightly across cohorts, reflecting cultural and methodological differences (Table S2 in the **Online Supplementary Document**). Questions ranged from binary assessments of hobby participation to comprehensive activity inventories covering volunteering, reading, games, *etc*. To ensure data comparability, we created a dichotomous hobby engagement variable (yes/no) for each cohort, where participants reporting engagement in any listed activities were classified as having hobby engagement, following previous research [17].

### Assessment of disability states

We evaluated functional disability status using standardised assessments of basic ADL and IADL across all study waves. We assessed six ADLs (dressing, walking across a room, bathing, eating, getting in or out of bed, and toileting) and six IADLs (meal preparation, shopping, telephone use, medication management, doing work around the house or garden, and money management). We operationalised disability states by incorporating both ADL and IADL, creating three distinct functional capacity levels following established methodological approaches: no disability (independent in all ADL and IADL), mild disability (difficulty with one or more IADLs while maintaining ADL independence), and severe disability (difficulty with one or more ADLs, regardless of IADL performance) [16,18]. This approach recognises that IADL limitations typically precede ADL impairments in the disablement process [16]. Death was included as an absorbing state in transition models. Due to sparse events of direct transitions from no disability to death (transition intensities ranging between 0.000006–0.0001 across Mexico, England, and China) and from mild disability to death (China transition intensity = 0.001), causing model non-convergence, we excluded these transitions from covariate-adjusted models, which is consistent with prior multi-state modelling research [19].

### Assessment of covariates

We selected covariates based on prior literature examining disability transitions and their potential to confound the association between hobby engagement and functional disability transitions. Sociodemographic characteristics included gender, age, education level, marital status, residence type, and living arrangement. Health-related behaviours comprised smoking and alcohol consumption history. We assessed health status by the presence of chronic conditions. Cognitive impairment was additionally included in MHAS and ELSA, as the other cohorts had substantial missing data for this variable. Socioeconomic factors included household income categorised into tertiles based on within-cohort distribution, except for CHARLS, where this variable was excluded due to substantial missing data. Depressive symptoms were excluded to avoid overadjustment bias, given bidirectional relationships with both exposure and outcome [3,20]. Physical activity was excluded as a hobby engagement measure in several cohorts because these cohorts included physical activity components (Table S3 in the **Online Supplementary Document**).

### Statistical analysis

Our statistical approach comprised two analytical components. First, multi-state Markov modelling of disability transition risks as the primary analysis, examining associations between hobby engagement and transition rates across functional states. And second, life expectancy estimation as an extended analysis, deriving expected years in different health states from the estimated transition parameters.

### Multi-state Markov modelling and parameter estimation

We employed continuous-time multi-state Markov models to analyse transitions between functional disability states and identify determinants of disability progression [21]. The models were implemented using the ‘msm’ package in *R*, version 4.3.2 (R Core Team, Vienna, Austria) [16], which enables estimation of transition rates between discrete states observed at irregular time intervals. Four states were defined: no disability, mild disability, severe disability, and death as an absorbing state. The models assumed that exact transition times were not directly observed, multiple transitions could occur between observation points, and death dates were recorded exactly [21]. Transition pathways were specified based on observed data patterns, with rare transitions excluded to ensure model stability, following established practices in previous multi-state modelling studies [19]. The multi-state modelling framework generated key analytical outputs, including HRs and 95% CIs for all covariates across transitions, representing instantaneous transition rates conditional on covariates. We present only covariate-adjusted HRs, as HRs are non-collapsible measures in which adjusted and unadjusted estimates may differ due to differences in covariate distributions rather than confounding alone. We evaluated the proportional-intensities assumption by fitting models that included interaction terms between hobby engagement and time. The vast majority of interactions were non-significant across all five cohorts, supporting the appropriateness of this assumption (Table S4 in the **Online Supplementary Document**). We estimated transition probabilities across states, with probabilities to disability and death calculated as competing-event probabilities rather than estimating the transition probability to disability conditional on being alive [16]. Mean sojourn times were computed to describe the average period spent in each state before transition [15].

### Extended analysis: life expectancy estimation

We estimated life expectancy measures using the ‘elect package’ in *R* [16], which fits multinomial regression models to estimate total and marginal life expectancy from multi-state model parameters [22]. Total life expectancy represents the expected remaining years of life at a given age. We calculated the marginal life expectancy for living states, representing the expected number of years spent in each state, regardless of initial state. Life expectancy measures were calculated at five-year age intervals from 50–90 years. We conducted stratified analyses by gender, residence type, and chronic disease status to evaluate the differential impacts of hobby engagement across population characteristics. Given comparisons at nine age points within each cohort and across stratified subgroups, we addressed multiple testing through cross-cohort replication rather than conventional statistical corrections. Associations observed in all five cohorts with consistent effect directions were considered robust, while cohort-specific findings were interpreted as exploratory. Stratified analyses were pre-specified as hypothesis-generating.

### Sensitivity analysis

To evaluate the robustness of our findings, we conducted several sensitivity analyses with different model specifications and sample restrictions. First, we examined models incorporating additional covariates, including cognitive impairment measures in China, the USA, and Europe, with household income additionally included in China, to assess potential residual confounding. Second, we simplified the disability classification to a three-state model (no disability, any disability, and death) to examine consistency across different disability categorisations. Third, we conducted analyses using ADL-only disability definitions, defining mild disability as having one ADL limitation and severe disability as having more than one ADL limitation, to evaluate whether the findings depended on the inclusion of IADL measures in the primary disability classification. Finally, we restricted analyses to participants with complete baseline covariates, while allowing missing values in exposure and time-varying covariates during follow-up (with disability states remaining complete) to assess whether patterns of missing data influenced the results. These sensitivity analyses confirmed the consistency of hobby engagement effects across different analytical approaches and strengthened confidence in the validity and generalisability of our findings. For hypothesis testing, we set significance at *P*-value <0.05.

## RESULTS

### Participant characteristics

Our analysis encompassed 127 650 participants aged ≥50 years from five major longitudinal studies – Mexico (n = 13 850), UK (n = 11 794), China (n = 16 261), USA (n = 15 938), and Europe (n = 69 807) (**Table 1**). Age distribution varied across regions, with participants aged 50–59 years predominating in UK and China, and those aged 60–69 years being the largest group in Mexico and Europe. Gender distribution showed a consistent female majority across all regions (50.9–58.1%). Regarding residential patterns, only the USA participants were predominantly urban, whereas China and Europe had higher proportions of rural dwellers. The vast majority of participants lived with others (78.2–94.1%) and were married or partnered (64.9–86.7%). Education level exhibited substantial variation. Mexico, China, UK, and European participants had predominantly less than upper-secondary education, while the USA had the highest proportion with tertiary education. Health behaviours varied considerably, with non-smokers ranging from 37.3% (UK) to 62.5% (Mexico), and alcohol consumption from 23.2% (Mexico) to 89.5% (UK). Chronic disease prevalence ranged from 45.2% (China) to 72.9% (USA). Cognitive impairment rates were similar in Mexico and the UK. Hobby engagement demonstrated pronounced regional variation: Europe exhibited the highest engagement (83.3%), followed by the UK (77.5%) and Mexico (68.8%), while China had the lowest (25.2%) and the USA showed moderate participation (53.1%).

**Table 1:** Basic demographics by cohort, n (%)

Variables	MHAS (n = 13 850)	ELSA (n = 11 794)	CHARLS (n = 16 261)	HRS (n = 15 938)	SHARE (n = 69 807)
Age in years					
*50–59*	4532 (32.7)	5243 (44.5)	8639 (53.1)	4950 (31.1)	21 612 (31.0)
*60–69*	5180 (37.4)	3386 (28.7)	4897 (30.1)	4439 (27.9)	24 215 (34.7)
*≥70*	4138 (29.9)	3165 (26.8)	2725 (16.8)	6549 (41.1)	23 980 (34.4)
Gender					
*Male*	7929 (57.2)	6226 (52.8)	8293 (51.0)	9237 (58.0)	38 651 (55.4)
*Female*	5921 (42.8)	5568 (47.2)	7968 (49.0)	6701 (42.0)	31 156 (44.6)
Residence					
*Rural*	NA	NA	9840 (60.5)	4845 (30.4)	47 232 (67.7)
*Urban*	NA	NA	6421 (39.5)	11 093 (69.6)	22 575 (32.3)
Live alone					
*No*	13 017 (94.0)	9219 (78.2)	15 304 (94.1)	12 397 (77.8)	55 826 (80.0)
*Yes*	833 (6.0)	2575 (21.8)	957 (5.9)	3541 (22.2)	13 981 (20.0)
Married					
*No*	4245 (30.6)	3190 (27.0)	2156 (13.3)	5632 (35.3)	17 011 (24.4)
*Yes*	9605 (69.4)	8604 (73.0)	14 105 (86.7)	10 306 (64.7)	52 796 (75.6)
Education level					
*Less than upper secondary education*	11 980 (86.5)	5108 (43.3)	14 268 (87.7)	2781 (17.4)	28 959 (41.5)
*Upper secondary and vocational training*	468 (3.4)	2298 (19.5)	1274 (7.8)	5556 (34.9)	25 926 (37.1)
*Tertiary education*	1402 (10.1)	4388 (37.2)	719 (4.4)	7601 (47.7)	14 922 (21.4)
Household income level					
*Low*	4960 (35.8)	3511 (29.8)	NA	4946 (31.0)	22 519 (32.3)
*Middle*	4237 (30.6)	3868 (32.8)	NA	5376 (33.7)	23 352 (33.5)
*High*	4653 (33.6)	4415 (37.4)	NA	5616 (35.2)	23 936 (34.3)
Smoke					
*No*	8651 (62.5)	4400 (37.3)	9533 (58.6)	6886 (43.2)	37 018 (53.0)
*Yes*	5199 (37.5)	7394 (62.7)	6728 (41.4)	9052 (56.8)	32 789 (47.0)
Drink					
*No*	10 632 (76.8)	1233 (10.5)	9489 (58.4)	7215 (45.3)	37 059 (53.1)
*Yes*	3218 (23.2)	10 561 (89.5)	6772 (41.6)	8723 (54.7)	32 748 (46.9)
Cognitive impairment					
*No*	11 612 (83.8)	10 089 (85.5)	NA	NA	NA
*Yes*	2238 (16.2)	1705 (14.5)	NA	NA	NA
Chronic disease					
*No*	5038 (36.4)	6033 (51.2)	8909 (54.8)	4312 (27.1)	28 918 (41.4)
*Yes*	8812 (63.6)	5761 (48.8)	7352 (45.2)	11 626 (72.9)	40 889 (58.6)
Hobby					
*No*	4324 (31.2)	2655 (22.5)	12 156 (74.8)	7477 (46.9)	11 673 (16.7)
*Yes*	9526 (68.8)	9139 (77.5)	4105 (25.2)	8461 (53.1)	58 134 (83.3)

CHARLS – China Health and Retirement Longitudinal Study, ELSA – English Longitudinal Study of Ageing, HRS – Health and Retirement Study, MHAS – Mexican Health and Ageing Study, NA – not available, SHARE – Survey of Health, Ageing and Retirement in Europe

### Transition probabilities

One-year transition probabilities varied considerably across countries (Table S5 in the **Online Supplementary Document**). The probability of remaining disability-free was highest in Europe and lowest in China. Recovery from mild disability to functional independence was substantial across all countries, ranging from 9.5% in the USA to 29.3% in China. Recovery from severe disability remained possible, with probabilities from 4.1% in the USA to 13.4% in Mexico. China showed the highest probabilities of deterioration from no disability to mild disability and from no disability to severe disability, whereas Mexico showed the lowest probability of deterioration from no disability to mild disability. Transitions from no disability to death remained consistently low across the four countries and Europe.

### Sojourn times

Mean sojourn times varied considerably across disability states and geographic regions (Table S6 in the **Online Supplementary Document**). In the no-disability state, participants spent 5.31 years in China, compared with over 12.15 years in Europe. The UK, Mexico, and the USA showed intermediate durations. Time spent in disability states was consistently shortest in China and longest in the USA, with mild disability ranging from 1.35–3.34 years and severe disability from 2.79–4.33 years. These findings highlight that regional health differences are most pronounced in disability-free longevity rather than disability duration.

### Hobby engagement and disability state transitions

Hobby engagement demonstrated consistent protective associations across multiple disability transitions (**Table 2**). The most robust finding was enhanced recovery from severe disability to functional independence, observed across all five regions with HR indicating 36.0–67.0% higher recovery rates, ranging from 1.36 (95% CI = 1.17, 1.57) in Mexico to 1.67 (95% CI = 1.34, 2.08) in the UK. Hobby engagement was also associated with reduced risk of disability onset, with 19.0–35.0% reductions in deterioration from no disability to mild disability observed in the four countries and Europe, with HR ranging from 0.65 (95% CI = 0.55, 0.76) in the USA to 0.81 (95% CI = 0.69, 0.96) in the UK. In contrast, deterioration from no disability to severe disability showed a 19.0% reduction only in the UK (HR = 0.81; 95% CI = 0.68, 0.95), with weaker associations in other countries. Additionally, hobby engagement was associated with 16.0–30.0% reduced mortality risk among those with severe disability in the four countries and Europe, with the strongest association observed in Europe (HR = 0.70; 95% CI = 0.64, 0.76) and the most modest in the USA (HR = 0.84; 95% CI = 0.76, 0.93). Recovery from mild disability and deterioration from mild-to-severe disability showed protective associations only in Europe, while other countries demonstrated weaker results for these transitions.

**Table 2:** Associations between hobby participation and disability transitions by cohort (adjusted)*

State transition	MHAS HR (95% CI)	*P*-value	ELSA HR (95% CI)	*P*-value	CHARLS HR (95% CI)	*P*-value	HRS HR (95% CI)	*P*-value	SHARE HR (95% CI)	*P*-value
State 1–state 2	0.66 (0.47, 0.92)	0.014	0.81 (0.69, 0.96)	0.017	0.71 (0.61, 0.84)	<0.001	0.65 (0.55, 0.76)	<0.001	0.66 (0.61, 0.72)	<0.001
State 1–state 3	0.87 (0.74, 1.03)	0.099	0.81 (0.68, 0.95)	0.011	0.89 (0.76, 1.04)	0.149	0.88 (0.77, 1.00)	0.055	1.02 (0.88, 1.18)	0.800
State 1–state 4	NA		NA		NA		0.81 (0.57, 1.16)	0.250	1.03 (0.74, 1.44)	0.855
State 2–state 1	1.16 (0.86, 1.57)	0.341	0.99 (0.81, 1.21)	0.932	1.128 (0.93, 1.34)	0.226	1.07 (0.90, 1.27)	0.464	1.19 (1.07, 1.32)	0.002
State 2–state 3	1.11 (0.85, 1.46)	0.446	0.94 (0.74, 1.19)	0.595	0.83 (0.64, 1.09)	0.180	0.84 (0.66, 1.07)	0.158	0.82 (0.73, 0.92)	0.001
State 2–state 4	2.24 (0.44, 11.48)	0.332	1.08 (0.80, 1.47)	0.600	1.44 (0.90, 2.30)	0.130	0.94 (0.72, 1.22)	0.631	1.48 (0.98, 2.24)	0.061
State 3–state 1	1.36 (1.17, 1.57)	<0.001	1.67 (1.34, 2.08)	<0.001	1.57 (1.30, 1.89)	<0.001	1.56 (1.27, 1.92)	<0.001	1.52 (1.33, 1.75)	<0.001
State 3–state 2	1.23 (0.94, 1.62)	0.129	0.87 (0.67, 1.11)	0.264	0.91 (0.68, 1.22)	0.537	1.12 (0.84, 1.49)	0.431	1.55 (1.30, 1.86)	<0.001
State 3–state 4	0.80 (0.70, 0.91)	<0.001	0.71 (0.57, 0.87)	0.001	0.76 (0.61, 0.96)	0.019	0.84 (0.76, 0.93)	<0.001	0.70 (0.64, 0.76)	<0.001

CI – confidence interval, CHARLS – China Health and Retirement Longitudinal Study, ELSA – English Longitudinal Study of Ageing, HR – hazard ratio, HRS – Health and Retirement Study, MHAS – Mexican Health and Ageing Study, NA – not available, SHARE – Survey of Health, Ageing and Retirement in Europe

*State 1 – functional independence, state 2 – mild disability, state 3 – severe disability, state 4 – death. All cohorts were adjusted for age, gender, living alone, marital status, education level, smoking, drinking, and chronic disease. HRS and SHARE further adjusted for household income level, while MHAS and ELSA additionally adjusted for household income level and cognitive function.

### Life expectancy

Hobby engagement was consistently associated with higher functional and total life expectancy across all five regions (**Table 3**). For functional life expectancy, hobby engagement was associated with differences ranging from 4.73–5.17 years at the age of 50 years to 0.61–0.93 years at the age of 90 years across the four countries and Europe, maintaining statistical significance across the entire age spectrum (*P* < 0.001). Total life expectancy showed similar patterns, ranging from 2.46–4.64 years at the age of 50 years to 0.46–1.09 years at the age of 90 years across the four countries and Europe. In contrast, associations between hobby engagement and disability duration showed heterogeneous patterns. For mild disability life expectancy, hobby engagement was associated with reduced time in the USA (ages 50–90, *P* < 0.05) and in China (ages 50–65, *P* < 0.05). For severe disability life expectancy, reductions were observed in Europe (ages 50–60, *P* < 0.05) and China (ages 50–75, *P* < 0.05), while Mexico and the UK showed predominantly non-significant associations. These findings were most pronounced at younger ages and gradually attenuated with advancing age, showing universal benefits for functional and total life expectancy, whereas disability duration showed limited, region-specific associations.

**Table 3:** Differences in life expectancy by hobby participation status across age groups and cohorts

Life expectancy and age	MHAS		ELSA		CHARLS		HRS		SHARE	
	**Difference**	***P*-value**	**Difference**	***P*-value**	**Difference**	***P*-value**	**Difference**	***P*-value**	**Difference**	***P*-value**
Functional life expectancy										
*50*	4.73	<0.001	5.17	<0.001	5.01	<0.001	4.95	<0.001	5.15	<0.001
*55*	4.20	<0.001	4.65	<0.001	4.49	<0.001	4.31	<0.001	4.73	<0.001
*60*	3.72	<0.001	4.03	<0.001	3.87	<0.001	3.61	<0.001	4.22	<0.001
*65*	3.12	<0.001	3.40	<0.001	3.25	<0.001	2.97	<0.001	3.65	<0.001
*70*	2.53	<0.001	2.79	<0.001	2.66	<0.001	2.33	<0.001	3.06	<0.001
*75*	2.00	<0.001	2.16	<0.001	2.12	<0.001	1.78	<0.001	2.46	<0.001
*80*	1.50	<0.001	1.60	<0.001	1.65	<0.001	1.32	<0.001	1.88	<0.001
*85*	1.09	<0.001	1.14	<0.001	1.26	<0.001	0.92	<0.001	1.37	<0.001
*90*	0.75	<0.001	0.74	<0.001	0.92	<0.001	0.61	<0.001	0.93	<0.001
Mild disability life expectancy										
*50*	0.14	0.448	–0.01	0.973	–1.05	<0.001	–0.88	<0.001	–0.09	0.341
*55*	0.13	0.437	0.03	0.863	–0.87	<0.001	–0.75	<0.001	–0.05	0.573
*60*	0.13	0.490	0.06	0.655	–0.70	0.001	–0.65	<0.001	–0.01	0.884
*65*	0.14	0.381	0.08	0.593	–0.53	0.012	–0.55	<0.001	0.04	0.629
*70*	0.14	0.403	0.10	0.484	–0.40	0.054	–0.46	<0.001	0.08	0.286
*75*	0.14	0.450	0.11	0.382	–0.32	0.107	–0.38	0.002	0.10	0.158
*80*	0.10	0.551	0.09	0.496	–0.23	0.213	–0.33	0.004	0.13	0.061
*85*	0.07	0.680	0.07	0.541	–0.18	0.320	–0.28	0.010	0.13	0.063
*90*	0.05	0.767	0.03	0.768	–0.15	0.289	–0.23	0.014	0.10	0.121
Severe disability life expectancy										
*50*	–0.45	0.300	–0.54	0.059	–1.50	<0.001	–0.08	0.652	–0.43	<0.001
*55*	–0.35	0.377	–0.37	0.141	–1.31	<0.001	–0.01	0.929	–0.35	0.004
*60*	–0.22	0.578	–0.17	0.499	1.11	<0.001	0.05	0.758	–0.24	0.033
*65*	–0.11	0.766	–0.02	0.948	–0.91	0.007	0.08	0.628	–0.15	0.145
*70*	–0.04	0.918	0.13	0.578	–0.74	0.024	0.11	0.479	–0.05	0.558
*75*	0.05	0.897	0.23	0.246	–0.61	0.041	0.13	0.375	0.03	0.744
*80*	0.05	0.887	0.29	0.145	–0.47	0.103	0.12	0.394	0.10	0.271
*85*	0.03	0.917	0.30	0.118	–0.38	0.109	0.10	0.406	0.11	0.211
*90*	–0.09	0.731	0.27	0.114	–0.32	0.099	0.07	0.530	0.05	0.512
Total life expectancy										
*50*	4.42	<0.001	4.63	<0.001	2.46	<0.001	3.99	<0.001	4.64	<0.001
*55*	3.99	<0.001	4.30	<0.001	2.31	<0.001	3.54	<0.001	4.34	<0.001
*60*	3.63	<0.001	3.93	<0.001	2.06	<0.001	3.01	<0.001	3.97	<0.001
*65*	3.16	<0.001	3.47	<0.001	1.81	0.001	2.50	<0.001	3.54	<0.001
*70*	2.63	<0.001	3.01	<0.001	1.52	0.006	1.98	<0.001	3.09	<0.001
*75*	2.18	<0.001	2.50	<0.001	1.19	0.016	1.52	<0.001	2.59	<0.001
*80*	1.65	<0.001	1.98	<0.001	0.95	0.036	1.12	<0.001	2.11	<0.001
*85*	1.20	0.004	1.51	<0.001	0.70	0.064	0.74	<0.001	1.61	<0.001
*90*	0.71	0.025	1.04	<0.001	0.46	0.083	0.46	0.011	1.09	<0.001

CHARLS – China Health and Retirement Longitudinal Study, ELSA – English Longitudinal Study of Ageing, HRS – Health and Retirement Study, MHAS – Mexican Health and Ageing Study, SHARE – Survey of Health, Ageing and Retirement in Europe

### Stratified analysis by gender, chronic disease status, and geographic location

Hobby engagement demonstrated consistent longevity benefits across demographic subgroups, though the magnitude varied by gender, health status, and geographic setting. Gender-specific patterns revealed notable regional heterogeneity. China showed a pattern in which males experienced greater total life expectancy gains, whereas Europe favoured females in total life expectancy, a pattern distinct from other regions, where benefits were generally comparable between genders. Functional life expectancy showed comparable benefits between genders across all regions. Chronic disease status emerged as a modifier of the benefits of hobby engagement in China, the UK, and Europe. In these regions, the absence of chronic disease was associated with greater gains in functional life expectancy than among those with chronic conditions. Other regions showed comparable benefits regardless of chronic disease status. Urban-rural analyses revealed geographic disparities primarily in China, while the USA and Europe showed minimal differences (Figure S6–31 in the **Online Supplementary Document**). Chinese urban residents had higher total life expectancy than their rural counterparts, whereas rural populations lost statistical significance for total life expectancy after the age of 60 years. For disability life expectancy in China and the USA, females showed greater reductions than males, and individuals without chronic conditions showed greater reductions than those with chronic conditions. Urban-rural differences were minimal.

### Sensitivity analyses

We conducted multiple sensitivity analyses to evaluate the robustness of the primary findings across different model specifications, covariate adjustments, missing-data strategies, and disability definitions. Two patterns demonstrated high consistency. First, hobby engagement was associated with reduced risk of functional deterioration across all analytical approaches: the transition from no disability to mild disability was significant in all five cohorts in primary analysis and remained significant after additional adjustment for cognitive function, with four of five cohorts showing significance both using ADL-only disability definitions and allowing follow-up missingness. Second, hobby engagement was associated with enhanced recovery from severe disability, though the specific recovery pathway varied by disability definition: recovery to functional independence was significant in all five cohorts in primary analysis, allowing follow-up missingness and after cognitive adjustment, while recovery to mild disability was significant in all five cohorts using ADL-only definitions. The simplified three-state model confirmed both reduced deterioration risk and enhanced recovery across all five cohorts. Associations with reduced mortality from severe disability were observed in all five cohorts in primary analysis and in the three-state model, though with attenuation in some cohorts after cognitive adjustment, allowing follow-up missingness and using ADL-only definitions. Other associations, including direct transition from no disability to severe disability, showed inconsistent patterns and should be interpreted with caution (Table S7–10 in the **Online Supplementary Document**).

## DISCUSSION

This comprehensive multi-state transition analysis across five major ageing cohorts provides robust evidence that hobby engagement is consistently associated with reduced functional state deterioration and promotes functional recovery across diverse global contexts. Hobby engagement was associated with 15.0–32.0% lower risk of worsening from functional independence to mild disability and 15.0–21.0% lower risk of worsening to severe disability across most regions, while universally enhancing recovery from severe disability by 38.0–70.0%. These associations translate into substantial differences in both functional and total life expectancy across the four countries and Europe, with functional life expectancy benefits ranging from 4.61–5.37 years and total life expectancy improvements of 2.83–4.87 years at the age of 50 years. These differences in life expectancy should be viewed as comparative expectations between individuals with and without hobby engagement, rather than as direct causal effects of hobby participation alone. These findings were validated through multiple sensitivity analyses. However, stratified analyses revealed substantial heterogeneity in benefit magnitude across demographic subgroups, suggesting that, while hobby engagement is a broadly applicable predictive marker of healthy ageing rather than a direct intervention, targeted implementation strategies may be needed to optimise its population-level impact.

The substantial cross-national disparities reveal critical insights into how socioeconomic development and healthcare infrastructure influence healthy ageing outcomes. China demonstrated a shorter mean sojourn time in the no-disability state and markedly higher transition probabilities for functional state worsening than in Western populations. These disparities reflect structural inequities in healthcare access, particularly in rural areas where 60.3% of CHARLS participants resided [23], where limited preventive care, early intervention services, and rehabilitation resources may accelerate functional decline [24]. The predominantly agricultural workforce faces additional challenges from cumulative occupational exposures that compromise functional capacity in later life [25]. These findings underscore the urgent need for targeted policy interventions, including strengthened rural healthcare infrastructure, enhanced occupational health protections, and expanded access to preventive services. This evidence provides critical justification for prioritising healthy ageing initiatives and healthcare equity investments in low- and middle-income countries, where rapid population ageing without corresponding healthcare system development threatens accelerated functional decline. Given these cross-national differences, implementation approaches may need to account for local healthcare infrastructure, community resources, and population characteristics.

Our results show that hobby engagement offers robust protection against transition from no disability to disability, significantly extending previous research [26,27] by demonstrating benefits across diverse individual pursuits and community-based activities. The underlying protective mechanisms operate through interconnected biological and social pathways that preserve functional capacity. Hobby engagement provides opportunities for physical functioning that reduce motor decline [28,29], maintain activities of daily living [30] and mobility [31], while delivering cognitive stimulation, social support, and a sense of purpose that preserve functional status [32]. Additionally, participation in social hobby activities contributes to improved mental health [33] and cardiovascular health [34], both of which are established risk factors for functional decline. The consistency of these protective associations across diverse populations suggests that hobby engagement engages fundamental healthy ageing mechanisms that operate independently of cultural or economic conditions. These observations point to several directions that warrant further exploration: clinicians could screen for hobby participation during routine geriatric assessments to identify individuals at elevated risk for functional decline; community health programs could offer hobby programs that may warrant exploration as potential interventions and provide evidence for integrating hobby-based activities at senior centres and libraries. These represent potential targets informed by our observational findings; experimental evaluation would be needed to determine their effectiveness.

The universal facilitation of recovery from severe disability across all populations is one of our most robust findings, with hobby engagement consistently associated with a 38.0–70.0% increase in the likelihood of recovery. Cognitively, hobby activities stimulate neuroplasticity through imagination, novelty, and creative processes, thereby enhancing cognitive reserve, which is essential for relearning daily living skills and developing coping strategies [4,35,36]. Physiologically, hobby activities often incorporate movement elements that strengthen physical capacity, including force, coordination, and cardiovascular fitness, crucial for functional independence, while addressing chronic conditions that hinder recovery [37–39]. The social dimensions provide support networks and practical assistance throughout recovery [40], while meaningful engagement fosters motivation and self-efficacy necessary for sustained rehabilitation [41]. Additionally, participation in hobbies offers positive emotional experiences that counteract psychological distress common in disability states, supporting adaptive coping strategies that enhance recovery outcomes [17]. Recovery from severe disability can meaningfully extend healthy life expectancy while reducing care dependency. In rehabilitation settings, several approaches merit investigation. Integrating patient-selected hobby activities into disability management protocols and establishing peer support networks connecting individuals with shared interests during recovery. However, we note that the observed associations may partly reflect socioeconomic advantages and psychosocial resources.

Hobby engagement was associated with reduced mild disability life years in the USA across all age groups, and in China at younger ages (50–65 years). The more consistent pattern in the USA may reflect distinctive advantages in the HRS measurement approach. The HRS employed a simple question asking whether respondents have a hobby or a pastime, which may capture personal identification and intrinsic motivation rather than specific behavioural activities. This approach allows individuals to self-define meaningful engagement, potentially distinguishing between activities pursued out of personal interest and those pursued for other reasons. Research suggests that all hobbies share common protective mechanisms regardless of activity type [17], and the subjective experience of personally meaningful activities may be more important than specific behaviours. Studies demonstrate that hobby engagement provides health benefits through multiple pathways [42], but these benefits may be strongest when activities are intrinsically motivated and personally valued rather than externally imposed. However, alternative explanations, including differences in population characteristics, healthcare systems, or unmeasured confounders, cannot be excluded. Future studies with standardised measurement approaches across populations are needed to clarify these patterns.

Several limitations should be acknowledged. First, despite employing previously validated measurement approaches and maintaining relative consistency across the five cohorts [5], variations in hobby engagement assessment methodologies existed. The USA and the UK used a single item asking whether participants had hobbies, whereas Mexico, China, and Europe inferred hobby engagement from participation in listed social activities with differing reference periods and without consistently capturing frequency or duration of engagement. These survey design constraints necessitated the use of a binary exposure measure and precluded construction of dose- or frequency-based indicators. As a result, individuals with minimal and intensive engagement are grouped together, and physical *vs.* non-physical activities cannot be clearly disentangled (*e.g.* ‘gone to a sport, social or other kind of club’), which also precludes identification of which specific activities are most beneficial, introducing non-differential misclassification that is likely to attenuate or obscure true associations and render our estimates conservative. Second, although sensitivity analyses using alternative disability definitions (simplified three-state model and ADL-only definitions) yielded consistent results, disability states assessed using ADL and IADL instruments may still be subject to misclassification, potentially biasing effect estimates toward the null. Third, separate models were fitted for each population without formal multiple testing correction, which may inflate Type one error rates. However, consistent protective associations across all five diverse populations make chance findings unlikely. Fourth, although sensitivity analyses that allowed for follow-up missingness produced consistent findings, our study adopted a complete-case analysis in accordance with prior research [10], excluding participants with missing covariate data at baseline or during follow-up. If missingness was non-random, this may introduce selection bias and limit generalizability to populations with complete data [43]. Fifth, although we adjusted for health status, socioeconomic factors, and social connectedness, residual confounding by unmeasured characteristics, such as personality traits and intrinsic resilience, cannot be excluded. In particular, individuals who are able to maintain hobby engagement despite severe disability may be inherently more resilient, potentially driving both continued engagement and recovery, so the observed associations may reflect a combination of direct benefits of hobby engagement and selection of more resilient individuals. Sixth, the continuous-time Markov model assumes that transition intensities depend only on the current state and covariates, not on the duration spent in that state. In our multi-cohort panel data, the exact duration of disability episodes cannot be determined due to interval censoring, precluding reliable estimation of duration-dependent semi-Markov models. This specification should be viewed as a pragmatic approximation; future studies with higher-frequency data may allow more flexible models. Furthermore, due to ELSA data constraints regarding mortality ascertainment after 2012, we utilised data spanning years 2002–2012 for ELSA, while the other four cohorts employed data from 2008 onwards, potentially introducing period effects and cohort-specific differences. Finally, no formal power calculation was conducted, as the sample size was determined by participant availability in each cohort. However, the large overall sample and replication across five independent populations provide confidence in the robustness of our findings.

## CONCLUSIONS

This multinational analysis of 127 650 older adults from five longitudinal studies identified consistent associations between hobby engagement and disability state transitions across diverse cultural contexts. Across four countries and Europe, hobby participation was associated with enhanced recovery from severe disability (36.0–67.0% higher recovery rates), 19.0–35.0% reduced risk of worsening from no disability to mild disability and 16.0–30.0% reduced mortality risk among individuals with severe disability. These associations corresponded to differences in functional life expectancy of 4.73–5.17 years and total life expectancy gains of 2.46–4.64 years at the age of 50 years, with attenuation but maintained statistical significance throughout the ageing trajectory.

While the observational design precludes causal inference and residual confounding cannot be ruled out, the consistency of these associations across diverse international populations suggests that hobby engagement may be a potentially modifiable behavioural factor relevant to healthy ageing. Future experimental or quasi-experimental studies are needed to determine whether interventions promoting hobby engagement can causally improve disability trajectories. Public health initiatives should prioritise developing and implementing culturally appropriate programs that facilitate access to hobbies across diverse communities, with particular attention to addressing socioeconomic and infrastructure barriers that may limit engagement among vulnerable populations.

Future research should employ quasi-experimental designs to establish causality, such as exploiting natural experiments including the opening of new community centres or policy changes affecting recreational program availability, using difference-in-differences or regression discontinuity approaches. Such studies should incorporate active control conditions to distinguish the effects of hobby engagement per se from those of general social contact or physical activity, and should examine whether benefits persist after accounting for baseline functional capacity and socioeconomic resources.

## Additional material


Online Supplementary Document
