# Supplementary material for: Hobby engagement, disability transitions, and life expectancy: a multinational longitudinal study
Source: J Glob Health. 2026 May 8;16:04127. doi: 10.7189/jogh.16.04127 (PMC13154335; doi:10.7189/jogh.16.04127)
Supplement: Online Supplementary Document [file jogh-16-04127-s001.pdf]

**Supplement to: Liu Q, Yang F. Hobby engagement, disability transitions, and life expectancy: a multinational longitudinal study. J Glob Health. 2026;16:04127.**

# Hobby engagement, disability transitions, and life expectancy: a multinational longitudinal study

**Table S1.** Outline of JoGH guideline items

**Supplementary Methods S1:** Study design and participants

**Figure S1.** Flow chart of participant inclusion and exclusion in the Mexican Health and Aging Study (MHAS)

**Figure S2.** Flow chart of participant inclusion and exclusion in the English Longitudinal Study of Ageing (ELSA)

**Figure S3.** Flow chart of participant inclusion and exclusion in the China Health and Retirement Longitudinal Study (CHARLS)

**Figure S4.** Flow chart of participant inclusion and exclusion in the Health and Retirement Study (HRS)

**Figure S5.** Flow chart of participant inclusion and exclusion in the Survey of Health, Ageing and Retirement in Europe (SHARE)

**Table S2.** Measurement of hobby engagement across five longitudinal cohorts

**Table S3.** Harmonized definitions for covariates across five longitudinal cohorts

**Table S4.** Proportional Intensities Assumption Test for Hobby Engagement (Hobby × Time Interaction)

**Table S5.** One-year Transition Probabilities Between Disability States by Cohort (Unadjusted)

**Table S6.** Mean Sojourn Times (95% CI) in Each Disability State by Cohort (Unadjusted)

**Figure S6.** Heatmap of Life Expectancy Differences by Hobby Participation Status Among Female Participants in the Mexican Health and Aging Study (MHAS)

**Figure S7.** Heatmap of Life Expectancy Differences by Hobby Participation Status Among Male Participants in the Mexican Health and Aging Study (MHAS)

**Figure S8.** Heatmap of Life Expectancy Differences by Hobby Participation Status Among Female Participants in the English Longitudinal Survey of Ageing (ELSA)

**Figure S9.** Heatmap of Life Expectancy Differences by Hobby Participation Status Among Male Participants in the English Longitudinal Survey of Ageing (ELSA)

**Figure S10.** Heatmap of Life Expectancy Differences by Hobby Participation Status Among Female Participants in the China Health and Retirement Longitudinal Study (CHARLS)

**Figure S11.** Heatmap of Life Expectancy Differences by Hobby Participation Status Among Male Participants in the China Health and Retirement Longitudinal Study (CHARLS)

**Figure S12.** Heatmap of Life Expectancy Differences by Hobby Participation Status Among Female Participants in the Health and Retirement Study (HRS)

**Figure S13.** Heatmap of Life Expectancy Differences by Hobby Participation Status Among Male Participants in the Health and Retirement Study (HRS)

**Figure S14.** Heatmap of Life Expectancy Differences by Hobby Participation Status Among Female Participants in the Survey of Health, Ageing and Retirement in Europe (SHARE)

**Figure S15.** Heatmap of Life Expectancy Differences by Hobby Participation Status Among Male Participants in the Survey of Health, Ageing and Retirement in Europe (SHARE)

**Figure S16.** Heatmap of Life Expectancy Differences by Hobby Participation Status Among Participants without Chronic Disease in the Mexican Health and Aging Study (MHAS)

**Figure S17.** Heatmap of Life Expectancy Differences by Hobby Participation Status Among Participants with Chronic Disease in the Mexican Health and Aging Study (MHAS)

**Figure S18.** Heatmap of Life Expectancy Differences by Hobby Participation Status Among Participants without Chronic Disease in the English Longitudinal Survey of Ageing (ELSA)

**Figure S19.** Heatmap of Life Expectancy Differences by Hobby Participation Status Among Participants with Chronic Disease in the English Longitudinal Survey of Ageing (ELSA)

**Figure S20.** Heatmap of Life Expectancy Differences by Hobby Participation Status Among Participants without Chronic Disease in the China Health and Retirement Longitudinal Study (CHARLS)

**Figure S21.** Heatmap of Life Expectancy Differences by Hobby Participation Status Among Participants with Chronic Disease in the China Health and Retirement Longitudinal Study (CHARLS)

**Figure S22.** Heatmap of Life Expectancy Differences by Hobby Participation Status Among Participants without Chronic Disease in the Health and Retirement Study (HRS)

**Figure S23.** Heatmap of Life Expectancy Differences by Hobby Participation Status Among Participants with Chronic Disease in the Health and Retirement Study (HRS)

**Figure S24.** Heatmap of Life Expectancy Differences by Hobby Participation Status Among Participants without Chronic Disease in the Survey of Health, Ageing and Retirement in Europe (SHARE)

**Figure S25.** Heatmap of Life Expectancy Differences by Hobby Participation Status Among Participants with Chronic Disease in the Survey of Health, Ageing and Retirement in Europe (SHARE)

**Figure S26.** Heatmap of Life Expectancy Differences by Hobby Participation Status Among Rural Participants in the China Health and Retirement Longitudinal Study (CHARLS)

**Figure S27.** Heatmap of Life Expectancy Differences by Hobby Participation Status Among Urban Participants in the China Health and Retirement Longitudinal Study (CHARLS)

**Figure S28.** Heatmap of Life Expectancy Differences by Hobby Participation Status Among Rural Participants in the Health and Retirement Study (HRS)

**Figure S29.** Heatmap of Life Expectancy Differences by Hobby Participation Status Among Urban Participants in the Health and Retirement Study (HRS)

**Figure S30.** Heatmap of Life Expectancy Differences by Hobby Participation Status Among Rural Participants in the Survey of Health, Ageing and Retirement in Europe (SHARE)

**Figure S31.** Heatmap of Life Expectancy Differences by Hobby Participation Status Among Urban Participants in the Survey of Health, Ageing and Retirement in Europe (SHARE)

**Table S7.** Associations between Hobby Participation and Disability Transitions by Cohort (Further Adjusted)

**Table S8.** Associations between Hobby Participation and Disability Transitions by Cohort (Three-State Model )

**Table S9.** Associations between Hobby Participation and Disability Transitions by Cohort (ADL-Based Definition )

**Table S10.** Associations between Hobby Participation and Disability Transitions by Cohort (Allowing Missing Follow-up Covariates)

**Table S11.** Comparison summary of baseline characteristics between the included participants and excluded participants in MHAS

**Table S12.** Comparison summary of baseline characteristics between the included participants and excluded participants in ELSA

**Table S13.** Comparison summary of baseline characteristics between the included participants and excluded participants in CHARLS

**Table S14.** Comparison summary of baseline characteristics between the included participants and excluded participants in HRS

**Table S15.** Comparison summary of baseline characteristics between the included participants and excluded participants in SHARE

Table S1. Outline of JoGH guideline items

| JoGH guideline item | Author's Response |
|---|---|
| 1. Please list all papers published by each co-author in previous 3 years that were based on secondary analysis of a big data repository | Guo, Y., & Yang, F. (2025). Hobby engagement and all-cause and cause-specific mortality risk among people aged 50 years and older in 19 countries. J Glob Health, 15, 04181. doi:10.7189/jogh.15.04181<br><br>The other co-authors have not published such studies during this period. |
| 2. Please explain the key elements of your study design and the use of the available datasets that make your study an original scientific contribution | Key elements of study design:<br><br>This study conducted a harmonized multinational longitudinal cohort analysis using five major Health and Retirement Study–family datasets (CHARLS, MHAS, ELSA, HRS, and SHARE) covering 127,650 adults aged ≥50 years from 24 countries. The key elements of the design are as follows:<br><br>(i) harmonization of hobby engagement measures across culturally diverse cohorts into a comparable binary exposure (yes/no), based on consistent reporting of participation in any hobby-type activities;<br><br>(ii) operationalization of functional disability as dynamic multi-state processes (no disability, mild disability, severe disability, and death), defined jointly by ADL and IADL limitations to reflect realistic disablement pathways;<br><br>(iii) estimation of continuous-time multi-state Markov models to quantify transition hazards between disability states (both deterioration and recovery) under irregular follow-up intervals;<br><br>(iv) translation of transition parameters into age-specific total and functional life expectancy differences from ages 50–90 using multi-state life table methods; |

| | |
|---|---|
| | (v) cross-national comparison and subgroup analyses (gender, chronic disease status, and residence) plus extensive sensitivity analyses to verify robustness across alternative disability definitions and model specifications.<br><br>Original scientific contribution through use of datasets:<br><br>Our study provides an original contribution by integrating hobby engagement, disability transitions, and life expectancy within one unified multi-state framework across five harmonized international aging cohorts. Prior research using these datasets has typically examined hobbies in relation to single outcomes (e.g., mortality, mental wellbeing, or cognition) or described disability transitions without linking them to hobby engagement. By leveraging comparable longitudinal measurements from diverse sociocultural settings, we are the first to show that hobby engagement is consistently associated with lower disability onset, greater recovery from severe disability, and reduced mortality-from-disability, and to demonstrate how these transition advantages accumulate into substantial functional and total life expectancy gains across countries. This multinational design therefore advances both methodological and substantive understanding of healthy aging by revealing the dynamic pathways through which hobby participation shapes longevity and functional survival. |
| 3. Please list all publications that addressed similar research questions in the same dataset and indicate where you cited them in your paper | 1. Marroig A. Transitions across states with and without difficulties in performing activities of daily living and death: a longitudinal comparison of ten European countries. Eur J Ageing. 2023;20(1):18. (This study has been cited in the Methods)<br><br>2. Mak HW, Noguchi T, Bone JK, Wels J, Gao Q, Kondo K, et al. Hobby engagement and mental wellbeing among people aged 65 years and older in 16 countries. Nat Med. 2023;29(9):2233-40. (This study has been cited in the Methods)<br><br>3. Huang W, Xiao Q, Li Z, Chen Y, Wang X, Liu Q. Positive association between hobby participation and objective and subjective cognition among adults aged 50 years and over in 24 |

| | |
|---|---|
| | countries. Social Science & Medicine. 2025;383:118487. (This study has been cited in the Discussion)<br><br>4. Guo Y, Yang F. Hobby engagement and all-cause and cause-specific mortality risk among people aged 50 years and older in 19 countries. J Glob Health. 2025;15:04181. (This study has been cited in the Introduction) |
| 4. Please explain how you addressed multiple testing through an appropriately rigorous statistical threshold and indicate this in the methods section | Our study involves comparisons between hobby engagement groups at nine age points within each cohort, as well as stratified analyses. The multi-state Markov model jointly estimates all transition intensities within a unified likelihood framework, which does not constitute independent multiple testing.<br><br>Rather than applying conventional corrections such as Bonferroni adjustment, we addressed multiple comparisons through cross-cohort replication. This approach is appropriate because: (1) our five cohorts represent independent populations, providing natural replication; (2) for life expectancy outcomes (functional and total life expectancy), all 45 cohort-age combinations showed significant associations in the same direction ($p < 0.01$), a pattern extremely unlikely to occur by chance; (3) conventional corrections assume a single dataset, whereas replication across independent samples provides stronger evidence.<br><br>For stratified analyses, we pre-specified these as exploratory investigations and interpreted results based on consistency of patterns rather than individual p-values.<br><br>We have added the following statement to the Methods section:<br><br>"Given comparisons at nine age points within each cohort and across stratified subgroups, we addressed multiple testing through cross-cohort replication rather than conventional statistical corrections. Associations observed in all five cohorts with consistent effect directions were considered robust. Stratified analyses were pre-specified as exploratory and interpreted based on pattern consistency." |

| | |
|---|---|
| 5. Please declare to what extent have AI chatbots been used in developing your paper and to which parts of the paper did they contribute | AI chatbots were used solely for language polishing, including improving grammar, clarity, and flow of expression. They were not involved in study conception, methodology, data processing, statistical analysis, interpretation of findings, or formulation of scientific conclusions. All scientific ideas, analyses, and decisions were independently developed and verified by the authors. |

Supplementary Methods S1: Study design and participants

The Mexican Health and Aging Study (MHAS)

The Mexican Health and Aging Study represents a comprehensive longitudinal investigation of adults aged 50 and older and their spouses living in both urban and rural areas throughout Mexico. Established in 2001 as the first longitudinal study of aging in Mexico with a broad socioeconomic perspective, MHAS was designed to prospectively evaluate the impact of disease on health, function, and mortality while employing protocols and survey instruments highly comparable to the U.S. Health and Retirement Study[1]. The 2001 baseline comprised a nationally representative sample of 15,186 individuals born in 1951 or earlier, selected from residents of both rural and urban areas using the National Employment Survey (Encuesta Nacional de Empleo, ENE) conducted by the Instituto Nacional de Estadística y Geografía (INEGI) in Mexico[2]. Households with at least one resident aged 50 years or older were eligible for inclusion, with random selection of one age-eligible individual per household, and automatic inclusion of spouses or partners regardless of age. The sampling strategy covered all 32 states of Mexico, with deliberate oversampling of households in six states accounting for 40% of all migrants to the USA. Sample refreshment occurred in 2012 with the addition of a representative sample from the 1952-1962 birth cohorts, and again in 2018 with cohorts born 1963-1968.

Data collection follows a biennial schedule with waves conducted in 2001, 2003, 2012, 2015, and 2018, utilizing face-to-face interviews with paper and pencil (2001, 2003) and Computer Assisted Personal Interviews (CAPI) from 2012 onwards[2]. The study demonstrates exceptional response rates, with baseline response of 91.8%, follow-up rates of 93.3% (2003) and 88.1% (2012), maintaining high data quality through minimal losses to follow-up[3]. Mortality surveillance is conducted through active tracking and next-of-kin interviews, with comprehensive documentation of deceased participants enabling robust mortality analyses[4]. Enhanced data collection includes anthropometric measures and biomarker collection from subsamples. External validation studies confirm sample representativeness against national census data[5]. MHAS has generated extensive research output, demonstrating its value as a premier resource for aging research in developing countries[6]. For this analysis, we utilized MHAS data to examine social participation and lifestyle factors among middle-aged and older Mexican adults. The study's longitudinal design and comprehensive health assessments make MHAS particularly suitable for investigating disability transitions and their relationship with behavioral factors among middle-aged and older adults.

For the current analysis, data from waves 3-5 of the MHAS (2012-2018) were utilized, as consistent methodology for measuring hobby

participation was only introduced from wave 3 onwards. The baseline sample comprised 23,362 individuals aged 50 years and older. Following exclusion of 596 participants with missing disability state information at any assessment wave, 382 individuals with absent hobby and covariate data, and 8,534 participants with availability of only single-wave disability data, the final analytical cohort contained 13,850 participants, among whom 1,792 experienced mortality during the observation period. Figure S1 illustrates the participant selection and exclusion process.

The English Longitudinal Study of Ageing (ELSA)

The English Longitudinal Study of Ageing represents a comprehensive panel investigation of men and women aged 50 and older residing in private households across England. Established in 2002 as a companion study to the Health and Retirement Study in the USA， ELSA was designed to document the multidimensional experience of aging in 21st century England through the collection of economic, social, psychological, cognitive, health, biological and genetic data[7]. The initial cohort comprised 11,391 core members recruited from participants in the Health Survey for England (HSE) conducted in 1998, 1999, and 2001[8]， representing individuals ranging from 50 to 100 years of age at baseline. The sampling strategy involved selection from HSE respondents

who met specific eligibility criteria: membership in a participating household with at least one person agreeing to follow-up, birth before March 1, 1952, and residence in a private English household during initial fieldwork[7]. Comparisons with national census data confirmed the sample's broad representativeness of the English population[9]. The study architecture incorporates biennial data collection through computer-assisted personal interviews and self-completion questionnaires, supplemented by nurse visits every four years for biomarker assessment and detailed functional measurements[7]. Sample refreshment occurred at wave 3 to maintain representation of individuals aged 50-53 years, with additional refreshment samples added at wave 4 (ages 50-75) and wave 6 (ages 50-55) to enhance analytical capacity. Response rates demonstrated robust participant engagement, with household response rates of 70% and individual response rates of 67% at baseline， while cross-sectional follow-up rates ranged from 73% to 82% across waves and conditional response rates from 73% to 82% [9]. Mortality surveillance is conducted through linkage to the NHS Central Register, ensuring comprehensive vital status tracking[7]. The study incorporates comprehensive weighting procedures, including cross-sectional and longitudinal weights to address differential non-response and calibrate to population distributions. Data collection encompasses detailed assessments of physical health, cognitive function, biomarkers, and behavioral factors, with physical examinations

conducted during nurse visits and linkage to administrative records including Hospital Episode Statistics and cancer registration data. For this analysis, we utilized ELSA data to examine social and civic participation patterns, including measures of volunteering and social and cultural participation activities[10]. The study's multidisciplinary framework, international harmonization capabilities, and comprehensive follow-up procedures position ELSA as a premier resource for investigating the complex relationships between lifestyle factors and health outcomes among middle-aged and older adults. Waves included in our study received ethical approvals from London Multi-Centre Research Ethics Committee (Wave 1: MREC/01/2/91, Wave 2: MREC/04/2/006, Wave 3: 05/MRE02/63), National Hospital for Neurology and Neurosurgery & Institute of Neurology Joint Research Ethics Committee (Wave 4: 07/H0716/48), Berkshire Research Ethics Committee (Wave 5: 09/H0505/124), NRES Committee South Central - Berkshire (Wave 6: 11/SC/0374).

For the current analysis, data from waves 1-6 of the ELSA (2002-2012) were employed, given that mortality information was accessible only through wave 6. The baseline sample comprised 17,659 individuals aged 50 years and older. Following exclusion of participants with missing disability state information at any assessment wave (229), those with absent hobby and covariate data (2,234), and individuals with availability

of only single-wave disability data (3,402), the final analytical cohort contained 11,794 participants, among whom 2,085 experienced mortality during the observation period. Figure S2 illustrates the participant selection and exclusion process.

The China Health and Retirement Longitudinal Study (CHARLS)

The China Health and Retirement Longitudinal Study (CHARLS) is a nationally representative longitudinal survey designed to examine health and economic adjustments to rapid population aging in China, targeting individuals aged 45 years and older and their spouses[11]. Harmonized with leading international research studies following the Health and Retirement Study (HRS) model to ensure cross-study comparability, CHARLS was initiated between June 2011 and March 2012, encompassing 17,708 individual participants from 150 county-level units across 28 provinces[12]. The study implements a sophisticated multistage stratified probability-proportional-to-size sampling design: initially, 150 county-level units were randomly selected from all county-level units excluding Tibet, stratified by geographic region, urban/rural classification, and per capita GDP[13]. Subsequently, three primary sampling units (PSUs) consisting of administrative villages in rural areas and neighborhoods in urban areas were selected within each county[12].

Following comprehensive mapping and listing operations to establish sampling frames, households with members aged 39 years or older were randomly selected, with one age-eligible individual chosen per household. When the selected person was 45 years or older, both the participant and spouse were interviewed using face-to-face computer-assisted personal interviews (CAPI)[11,12]. The baseline survey achieved a response rate of 80.5%, with nonresponse attributed to refusal (8.8%), inability to contact residents (8.2%), and other reasons (2.0%)[12]. CHARLS employs biennial follow-up surveys with participants tracked in 2013, 2015, 2018, and 2020, incorporating proxy interviews when original respondents were unavailable due to migration or illness[13]. In cases where participant mortality was reported during subsequent follow-up waves, research teams attempted to locate knowledgeable family members to conduct exit interviews for obtaining mortality-related information[12]. The comprehensive data collection encompasses demographics, health status and functioning, physician-diagnosed chronic illnesses, lifestyle behaviors, cognitive assessment, depression screening, healthcare utilization, employment history, household economics, and family dynamics, supplemented by anthropometric measurements, physical performance tests, and biomarker collection[11]. To ensure data quality and address potential bias, CHARLS implements carefully constructed sampling weights that correct for nonresponse and sampling-

frame errors at each methodological stage[12,13]. The study was approved by the Biomedical Ethics Committee of Peking University (IRB00001052-11015).

For the current analysis, data from waves 1-4 of the CHARLS (2011-2018) were utilized. There were 22,386 participants aged 50 and over at baseline. We further excluded 733 participants due to missing disability state information at any assessment wave, 461 individuals with absent hobby and covariate data, and 4,931 participants with availability of only single-wave disability data, the final analytical cohort contained 16,261 participants, of whom 1,630 died during the follow-up period. Figure S4 depicted participant inclusion and exclusion.

The Health and Retirement Study (HRS)

The Health and Retirement Study represents a biennial longitudinal investigation of aging Americans, encompassing over 37,000 participants aged 50 and older across 23,000 households nationwide. Initiated in 1992, this study employs a multidisciplinary framework examining health, economic status, employment transitions, and family dynamics throughout the aging process[14]. The sampling methodology utilizes a complex multi-stage area probability design with geographic stratification and deliberate oversampling of African-American and Hispanic

populations at twice the rate of White participants to ensure adequate minority representation[15]. The study population was constructed through sequential cohort recruitment, beginning with the original 1931-1941 birth cohort in 1992, followed by additional cohorts enrolled in 1998 (Children of Depression and War Babies), 2004 (Early Baby Boomers), and 2010 (Mid Baby Boomers) to maintain population representativeness. Data collection employs a mixed-mode approach where alternating half-samples receive either enhanced face-to-face interviews including physical measurements and psychosocial assessments, or core telephone interviews, ensuring comprehensive data availability every four years for each participant. Response rates remain consistently high, with follow-up rates exceeding 85% across waves and baseline participation ranging from 69.9% to 81.6% depending on cohort[14].

Vital status monitoring combines active tracking efforts with National Death Index linkages, achieving near-complete mortality ascertainment[14]. Sample weights account for complex sampling design and differential response patterns, enabling population-level inferences[16]. The latest ethical approval for HRS was obtained from the University of Michigan Institutional Review Board (IRB Protocol: HUM0061128).

For this analysis, we utilized HRS data to assess hobby participation through survey questions covering activities including volunteering, organizational membership, reading, and recreational games[10]. Data from waves 9-15 of the HRS (2008-2020) were utilized, as wave 9 marked the introduction of consistent hobby engagement measurements. Functional disability assessment employed standardized measures of activities of daily living (ADL) and instrumental activities of daily living (IADL) collected through the core interview protocol. The longitudinal design and comprehensive health assessments make HRS particularly suitable for examining disability transitions and their relationship to lifestyle factors among middle-aged and older adults. The initial sample included 31,033 participants aged 50 years and older at baseline. After excluding individuals with missing disability state information across assessment waves (199), participants with incomplete hobby and covariate information (5,610), and those providing only single-wave disability data (9,286), the final analytic sample comprised 15,938 participants, of whom 5,334 died during the follow-up period. Figure S4 demonstrates the participant inclusion and exclusion procedures.

The Survey of Health, Ageing and Retirement in Europe (SHARE)

The Survey of Health, Ageing and Retirement in Europe (SHARE) represents a comprehensive multidisciplinary longitudinal study examining individuals aged 50 years and older across European countries and Israel, designed to provide infrastructure for understanding the individual and population aging process[17,18]. Established as a response to the European Commission's interest in obtaining scientific evidence on population aging, SHARE was created as a longitudinal survey infrastructure by researchers from multiple disciplines following its development initiation in 2002[17]. Since its inception in 2004, SHARE has expanded from its initial 11 participating countries to encompass 28 European countries and Israel, evolving into a unique panel database of micro data covering health, socioeconomic status, and social and family networks. The study is closely modeled after and harmonized with the US Health and Retirement Study (HRS) and the English Longitudinal Study of Ageing (ELSA), establishing itself as a role model for aging surveys worldwide[17,18]. SHARE employs sophisticated multistage stratified probability sampling approaches that divide countries into strata representing different geographical areas, achieving an average response rate of 62% at the household level in its initial baseline wave that utilized over 50,000 addresses across participating countries[17]. Data collection occurs biennially through face-to-face computer-assisted personal interviewing (CAPI), supplemented by self-administered questionnaires,

with comprehensive assessment covering demographics, health status, socioeconomic circumstances, behavioral risks, cognitive function, employment history, and social networks across 20 questionnaire modules[17]. The study has incorporated innovative methodological features including retrospective life history data collection through SHARELIFE and end-of-life interviews with proxy respondents for deceased participants, which have been validated as appropriate sources of information for mortality studies[19]. SHARE maintains strict ex-ante harmonization across all participating countries, ensuring international comparability while accommodating diverse European welfare state regimes and country-specific circumstances in sampling procedures[18]. Physical measurements and biomarker collection have been progressively incorporated across waves to complement self-reported health data and provide objective health assessments[17]. The research infrastructure supports extensive multidisciplinary comparative research on aging processes, with researchers worldwide utilizing SHARE data for their studies across various disciplines. The SHARE was approved by the Ethics Committee of the University of Mannheim and Ethics Council of the Max Planck Society (00006320).

For the current analysis, consistent with previous research[10], data from waves 4-8 of the SHARE (2010-2019) were utilized, as wave 4 started collecting questions about hobby engagement. The initial sample

included 127,482 participants aged 50 years and older at baseline. We further excluded 1,083 participants due to missing disability state information at any assessment wave, 12,615 individuals with baseline hobby and covariate data, and 43,977 participants with availability of only single-wave disability data, the final analytical cohort contained 69,807 participants, of whom 10,386 died during the follow-up period. Figure S5 depicted participant inclusion and exclusion. The sample sizes for each country in the final analysis were as follows: Austria (n = 4,202), Germany (n = 4,519), Sweden (n = 3,893), Netherlands (n = 2,915), Spain (n = 5,665), Italy (n = 4,617), France (n = 4,757), Denmark (n = 3,873), Gresce (n = 3,532), Switzerland (n = 3,067), Belgium (n = 5,609), Israel (n =1,619), Czech Republic (n = 5,364), Poland (n = 1,519), Luxembourg (n = 1,329), Hungary (n = 891), Portugal (n = 1,297), Slovenia (n = 3,716), Estonia (n = 6,248), and Croatia (n = 1,175).

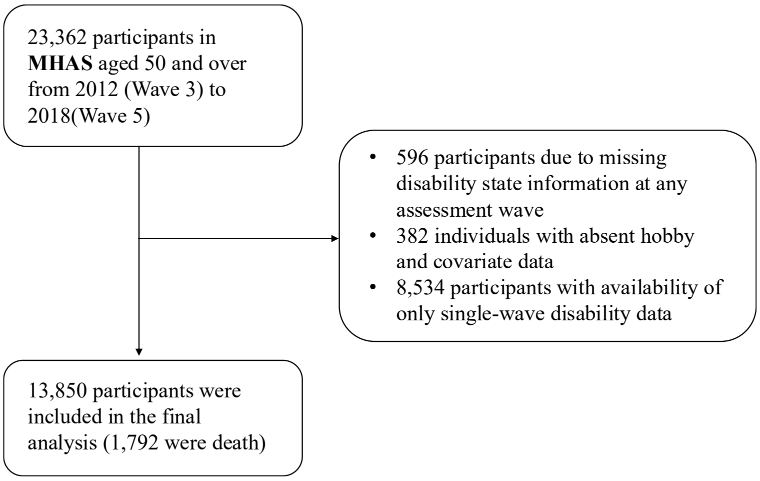


**Figure S1.** Flow chart of participant inclusion and exclusion in the Mexican Health and Aging Study (MHAS)

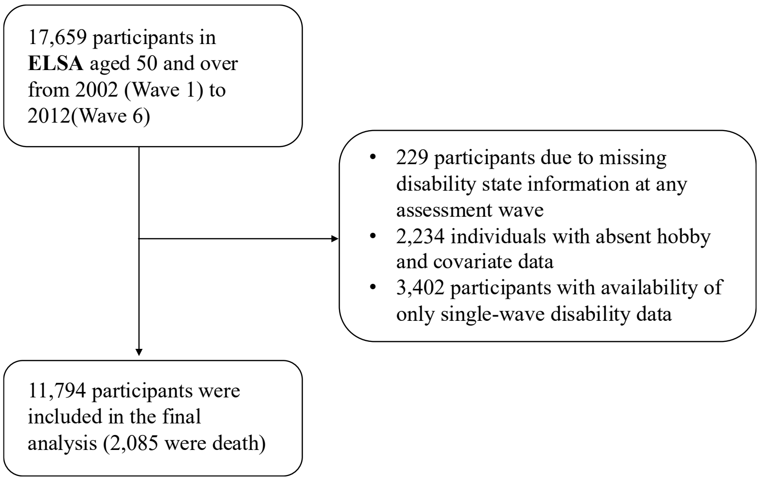


**Figure S2.** Flow chart of participant inclusion and exclusion in the English Longitudinal Study of Ageing (ELSA)

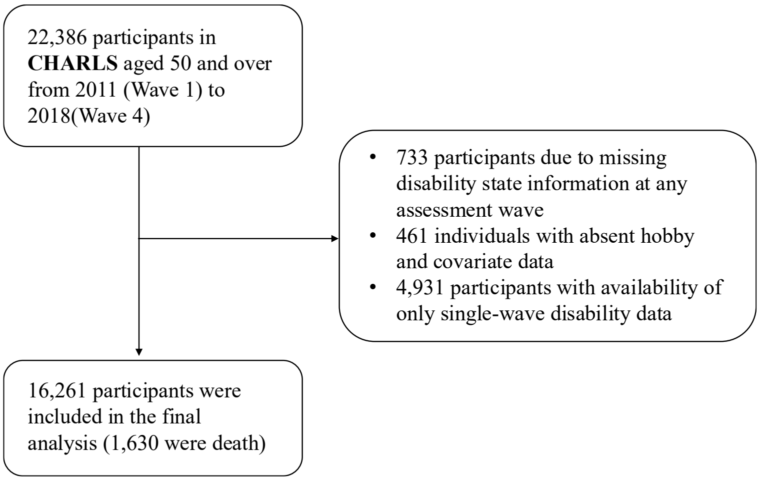


**Figure S3.** Flow chart of participant inclusion and exclusion in the China Health and Retirement Longitudinal Study (CHARLS)

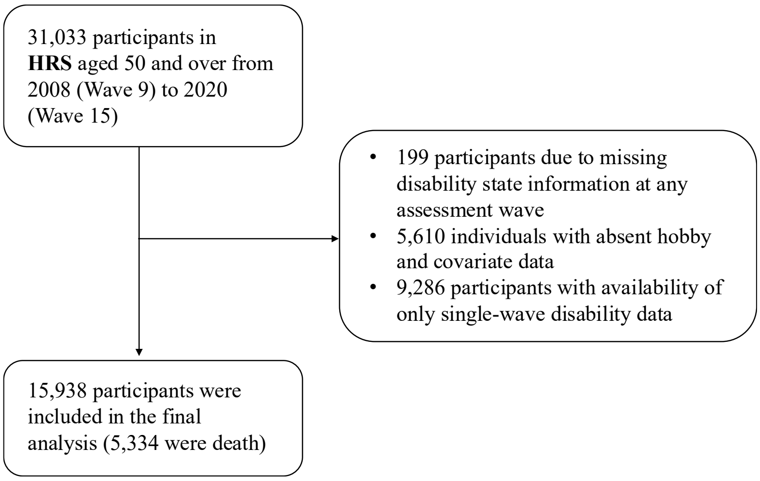


**Figure S4.** Flow chart of participant inclusion and exclusion in the Health and Retirement Study (HRS)

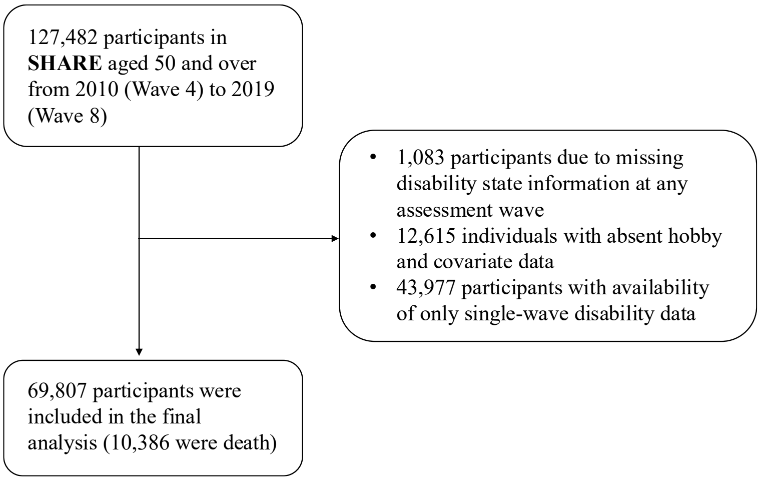


**Figure S5.** Flow chart of participant inclusion and exclusion in the Survey of Health, Ageing and Retirement in Europe (SHARE)

**Table S2.** Measurement of hobby engagement across five longitudinal cohorts

| Survey cohort | Questions | Cut-off value | Reference |
|---|---|---|---|
| MHAS | 1. Does respondent volunteer/support an organization without pay/reward? 2. Does respondent attend a training course? 3. Does respondent attend a sporting/social club? 4. Does respondent read books/magazines/a newspaper? 5. Does respondent do crossword puzzles/jigsaw puzzles/Sudoku? 6. Does respondent play games? | 1=any of the activities; 0=none | Mak, H.W., Noguchi, T., Bone, J.K. et al. Hobby engagement and mental wellbeing among people aged 65 years and older in 16 countries. Nat Med 29, 2233–2240 (2023). https://doi.org/10.1038/s41591-023-02506-1 |
| ELSA | How often do you work on a hobby or project? | 1=any frequency; 0=not in the last month/never | |
| CHARLS | Which of the social activities listed on this card – if any – have you done in the past month?’ 1. Played ma-jong, played chess, played cards or went to a community club. 2. Went to a sport, social or other kind of club. 3. Took part in a community-related organization. 4. Did voluntary or charity work. 5. Attended an educational or training course. 0. None of these | 1=any of the activities; 0=none | |
| HRS | I have a hobby or past time. | 1=yes; 0=no | |
| SHARE | Which of the activities listed on this card – if any – have you done in the past twelve months?’ 1. Done voluntary or charity work. 2. Attended an educational or training course. 3. Gone to a sport, social or other kind of club. 4. Taken part in a political or community-related organization. 5. Read books, magazines or newspapers. 6. Did word or number games such as crossword puzzles or Sudoku. 7. | 1=any of the activities; 0=none | |

| | | | | |
|---|---|---|---|---|
| | Played cards or games such as chess. 0. None of these | | | |

Note: *MHAS – Mexican Health and Aging Study; ELSA – English Longitudinal Study of Ageing; CHARLS – China Health and Retirement Longitudinal Study; HRS – Health and Retirement Study; SHARE – Survey of Health, Ageing and Retirement in Europe.

**Table S3.** Harmonized definitions for covariates across five longitudinal cohorts

| **Variables** | **Harmonized values** | **Definition in five surveys** | | | | |
|---|---|---|---|---|---|---|
| | | ELSA | HRS | SHARE | CHARLS | MHAS |
| Age[20] | 1 = 50-59 years; 2 = 60-69 years; 3 = 70 years or greater | | | | | |
| Gender[10] | 1= men;<br>0= women | The gender was reported as men or women. | | | | |
| Residence[21] | 1= urban; 0= rural | \ | The residence was reported rural or urban. | | | \ |
| Live alone[22] | 1= yes | The number of household members is one. | | | | |
| | 0= no | The number of household members greater than one. | | | | |
| Education level[23] | 1= Less than lower secondary education | Less than high school. | | None, Primary education, or lower secondary education. | "No Formal Education (Illiterate)", "Did Not Finish Primary School but can Read", "Sishu (Private Tutoring)", | Less than primary education,Primary education, Lower secondary education |

| | | | | | | |
|---|---|---|---|---|---|---|
| | | | | | "Elementary School" or "Middle School". | |
| | 2= Upper secondary and vocational training | High-school graduate, or some college. | GED, high-school graduate, or some college. | Upper secondary education, post-secondary non tertiary education. | "High School" or "Vocational School". | Upper secondary education |
| | 3= Tertiary education | College and above. | | First stage of tertiary education, or Second stage of tertiary education. | "Two/three-year College", "College Grad" or "Post-graduate degree". | First stage of tertiary education, Second stage of tertiary education |

| | | | | | |
|---|---|---|---|---|---|
| Marital status[24] | 1= married/partnered | Married and living together with spouse; Partnered. | | | |
| | 0= single | Separated, divorced, widowed, and never married. | | | |
| Household income level[25] | 1= lowest; 2 = middle; 3 = highest | Household income was divided into tertiles using the rank function, from lowest (1) to highest (3). | | | |
| Smoke[26] | 1= Smoker | Participants ever smoked cigarettes. | | | |
| | 0= Non-smoker | Participants has never smoked. | | | |
| Drink[23] | 1= Yes | Participants had consumed alcohol in the past 12 months. | Participants had previously consumed alcohol. | Participants drank alcohol or more weekly. | Participants had previously consumed alcohol. |
| | 0= No | Participants had not consumed alcohol in the past 12 months. | Participants never drunk alcoholic. | Participants drank alcohol less than weekly. | Participants never drunk alcoholic. |

| | | | | | |
|---|---|---|---|---|---|
| Cognitive impairment[27] | | immediate word recall (10) + delayed word recall (10) + orientation (4) = total score (24) | immediate word recall (10) + delayed word recall (10) + orientation (4) + serial 7's (5) = total score (29) | immediate word recall (10) + delayed word recall (10) + orientation (5) + serial 7's (5) + draw (1) = total score (31) | immediate word recall (8) + delayed word recall (8) + orientation (3) + serial 7's (5) = total score (24) |
| | 1=Yes | Participants were classified as having cognitive impairment if their cognitive score fell at least 1 standard deviation (SD) below age-specific norms. All participants aged 50 and above were grouped in five-year age brackets. | | | |
| | 0=No | Participants were classified as having normal cognition if their cognitive scores did not fall 1 SD or more below age-specific norms. | | | |
| Presence of chronic illness[28] | 1=1 or more disease | Participants had been diagnosed with chronic conditions listed below by a doctor: cancer, heart problems, stroke, diabetes, lung disease, or hypertension. | | | |
| | 0= None | Participants do not have any chronic diseases. | | | |

Note: *MHAS – Mexican Health and Aging Study; ELSA – English Longitudinal Study of Ageing; CHARLS – China Health and Retirement Longitudinal Study; HRS – Health and Retirement Study; SHARE – Survey of Health, Ageing and Retirement in Europe. GED – General Educational Development.

†Cognition test consists of five components, including immediate and delayed word recall, orientation, serial 7's and draw. For word recall, participants are required to recite 10 words in HRS, CHARLS, SHARE, and ELSA, and 8 words in MHAS. For orientation, participants are

asked whether they could remember the date of that day (day of week, day of month, month, and year in HRS, CHARLS, SHARE, and ELSA, and day of month, month, and year in MHAS), with CHARLS additionally including the season. For serial 7's, participants are required to make five calculations and answer how much is 100 minus 7. However, serial 7's test could be unavailable in ELSA 2002-2012 and MHAS 2012, so we only did not include the component in these waves. One point is given for each right answer, and the total score refers to the theoretical maximum score of all tests.

**Table S4.** Proportional Intensities Assumption Test for Hobby Engagement (Hobby × Time Interaction)

| State Transition | MHAS HR(95%CI) | ELSA HR(95%CI) | CHARLS HR(95%CI) | HRS HR(95%CI) | SHARE HR(95%CI) |
|---|---|---|---|---|---|
| State 1 - State 2 | 0.83 (0.68, 1.01) | 1.00 (0.94, 1.06) | 0.97 (0.88, 1.08) | 1.04 (0.98,1.11) | 1.03 (0.99, 1.07) |
| State 1 - State 3 | 1.14* (1.04, 1.25) | 0.95 (0.90, 1.00) | 1.05 (0.94, 1.17) | 0.95 (0.91,1) | 1.05 (0.98, 1.12) |
| State 1 - State 4 | \ | \ | \ | 0.83 (0.68,1.02) | 0.96 (0.84, 1.11) |
| State 2 - State 1 | 1.08 (0.84, 1.37) | 0.99 (0.92, 1.06) | 0.93 (0.82, 1.06) | 0.96 (0.9,1.03) | 1.00 (0.95, 1.05) |
| State 2 - State 3 | 0.97 (0.75, 1.25) | 1.01 (0.93, 1.10) | 0.88 (0.73, 1.06) | 1.03 (0.94,1.14) | 0.94* (0.90, 0.99) |
| State 2 - State 4 | 0.80 (0.45, 1.44) | 0.98 (0.88, 1.10) | 0.85 (0.65, 1.11) | 0.97 (0.86,1.1) | 1.17 (0.97, 1.41) |
| State 3 - State 1 | 1.04 (0.95, 1.15) | 0.92* (0.85, 0.99) | 1.06 (0.91, 1.24) | 0.98 (0.91,1.06) | 1.01 (0.95, 1.06) |
| State 3 - State 2 | 1.07 (0.77, 1.49) | 1.06 (0.97, 1.15) | 0.90 (0.72, 1.13) | 1.00 (0.89,1.11) | 1.07 (0.99, 1.16) |
| State 3 - State 4 | 1.08 (0.93, 1.26) | 0.95 (0.88, 1.03) | 1.08 (0.93, 1.24) | 1.00 (0.96,1.04) | 0.99 (0.96, 1.02) |

Note: *MHAS – Mexican Health and Aging Study; ELSA – English Longitudinal Study of Ageing; CHARLS – China Health and Retirement Longitudinal Study; HRS – Health and Retirement Study; SHARE – Survey of Health, Ageing and Retirement in Europe. HR – Hazard ratios; CI – Confidence Interval.

†State 1 = functional independence; State 2 = mild disability; State 3 = severe disability; State 4 = death. * indicates 5% significant ratio; \ indicates that this transition has not been incorporated into the multi-state model, as it is rarely observed.

‡All cohorts were adjusted for age, gender, live alone, marital status, education level, smoke, drink, and chronic disease. HRS and SHARE further adjusted for household

income level, while MHAS and ELSA additionally adjusted for household income level and cognitive function.

**Table S5.** One-year Transition Probabilities Between Disability States by Cohort (Unadjusted)

| State Transition | MHAS | ELSA | CHARLS | HRS | SHARE |
|---|---|---|---|---|---|
| state1-state1 | 0.9204 | 0.9202 | 0.8511 | 0.9221 | 0.9278 |
| state1-state2 | 0.0171 | 0.0357 | 0.0816 | 0.0295 | 0.0393 |
| state1-state3 | 0.0587 | 0.0400 | 0.0644 | 0.0317 | 0.0270 |
| state1-state4 | 0.0039 | 0.0042 | 0.0029 | 0.0168 | 0.0059 |
| state2-state1 | 0.1772 | 0.1973 | 0.2929 | 0.0951 | 0.2003 |
| state2-state2 | 0.4920 | 0.5226 | 0.5064 | 0.7448 | 0.5904 |
| state2-state3 | 0.2774 | 0.1727 | 0.1845 | 0.0935 | 0.1504 |
| state2-state4 | 0.0534 | 0.1075 | 0.0162 | 0.0666 | 0.0590 |
| state3-state1 | 0.1339 | 0.0989 | 0.1233 | 0.0408 | 0.0928 |
| state3-state2 | 0.0369 | 0.0742 | 0.1006 | 0.0359 | 0.0739 |
| state3-state3 | 0.7351 | 0.7580 | 0.7183 | 0.7967 | 0.7169 |
| state3-state4 | 0.0941 | 0.0689 | 0.0578 | 0.1266 | 0.1165 |

Note: *MHAS – Mexican Health and Aging Study; ELSA – English Longitudinal Study of Ageing; CHARLS – China Health and Retirement Longitudinal Study; HRS – Health and Retirement Study; SHARE – Survey of Health, Ageing and Retirement in Europe.
†State 1 = functional independence; State 2 = mild disability; State 3 = severe disability; State 4 = death.

**Table S6.** Mean Sojourn Times (95% CI) in Each Disability State by Cohort (Unadjusted)

| | MHAS | ELSA | CHARLS | HRS | SHARE |
|---|---|---|---|---|---|
| State 1 | 11.07 (10.66,11.49) | 10.98 (10.6,11.36) | 5.31 (5.13,5.49) | 11.95 (11.59,12.31) | 12.15 (11.94,12.36) |
| State 2 | 1.37 (1.23,1.53) | 1.48 (1.4,1.57) | 1.35 (1.28,1.43) | 3.34 (3.17,3.51) | 1.82 (1.77,1.87) |
| State 3 | 3.07 (2.90,3.25) | 3.40 (3.26,3.56) | 2.79 (2.68,2.91) | 4.33 (4.17,4.50) | 2.88 (2.81,2.96) |

Note: *MHAS – Mexican Health and Aging Study; ELSA – English Longitudinal Study of Ageing; CHARLS – China Health and Retirement Longitudinal Study; HRS – Health and Retirement Study; SHARE – Survey of Health, Ageing and Retirement in Europe.
†State 1 = functional independence; State 2 = mild disability; State 3 = severe disability.
‡Values are mean sojourn times in years with 95% confidence intervals in parentheses.

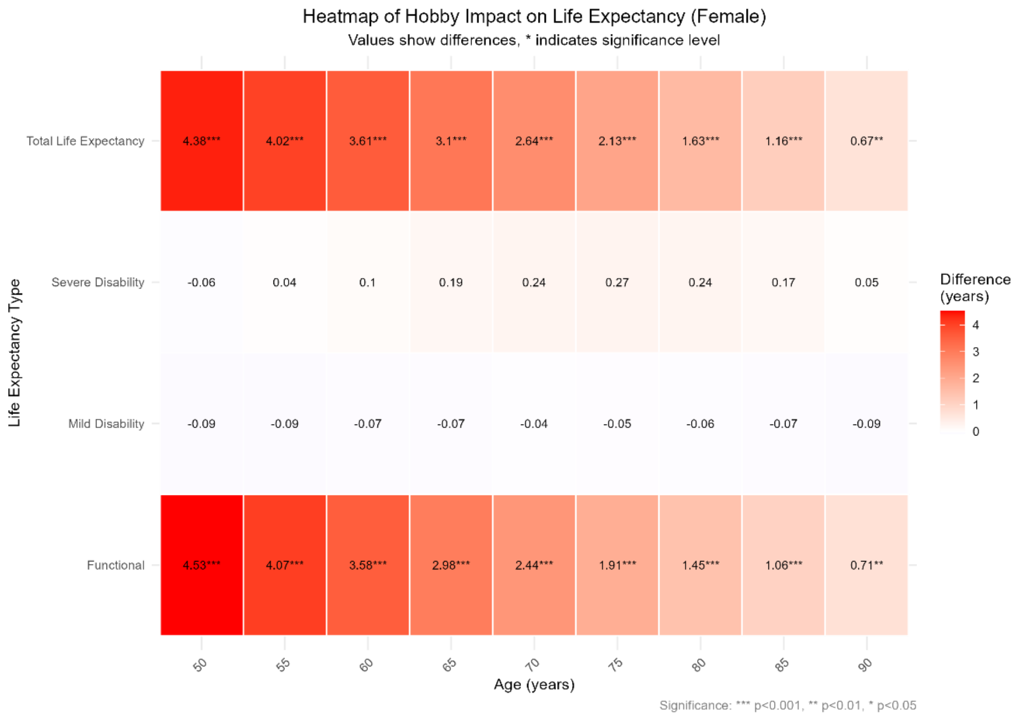


**Figure S6.** Heatmap of Life Expectancy Differences by Hobby

Participation Status Among Female Participants in the Mexican Health and Aging Study (MHAS). Values represent differences in life expectancy (years). * indicate statistical significance levels: *** p<0.001, ** p<0.01, * p<0.05

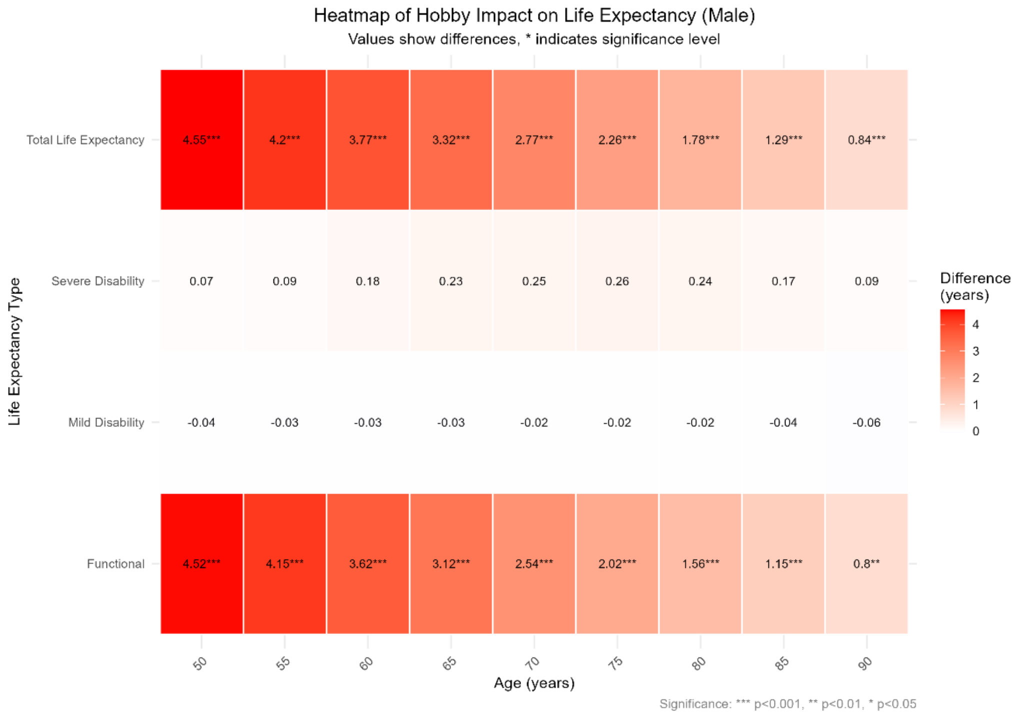

**Figure S7.** Heatmap of Life Expectancy Differences by Hobby

Participation Status Among Male Participants in the Mexican Health and Aging Study (MHAS). Values represent differences in life expectancy (years). * indicate statistical significance levels: *** p<0.001, ** p<0.01, * p<0.05

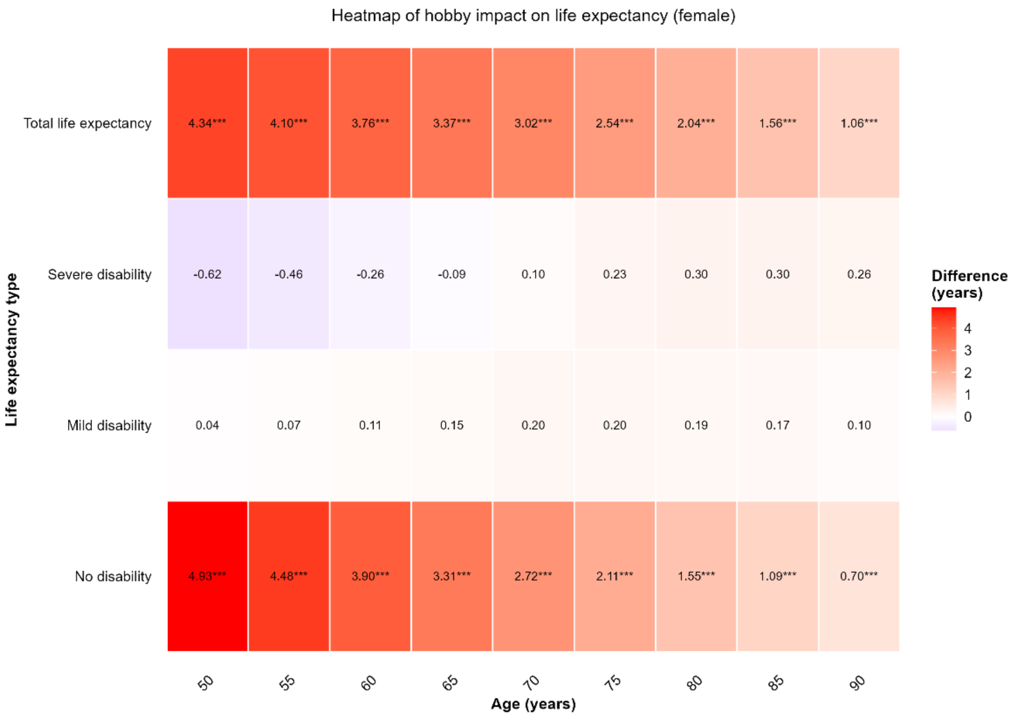


**Figure S8.** Heatmap of Life Expectancy Differences by Hobby

Participation Status Among Female Participants in the English Longitudinal Survey of Ageing (ELSA). Values represent differences in life expectancy (years). * indicate statistical significance levels: *** p<0.001, ** p<0.01, * p<0.05

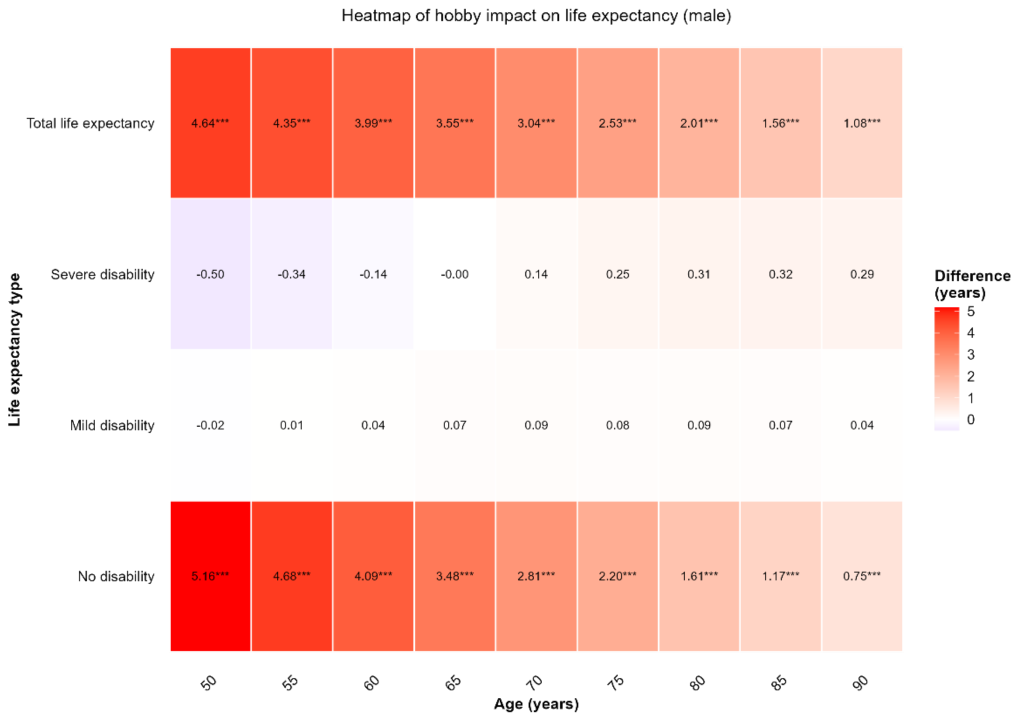


**Figure S9.** Heatmap of Life Expectancy Differences by Hobby

Participation Status Among Male Participants in the English Longitudinal Survey of Ageing (ELSA). Values represent differences in life expectancy (years). * indicate statistical significance levels: *** p<0.001, ** p<0.01, * p<0.05

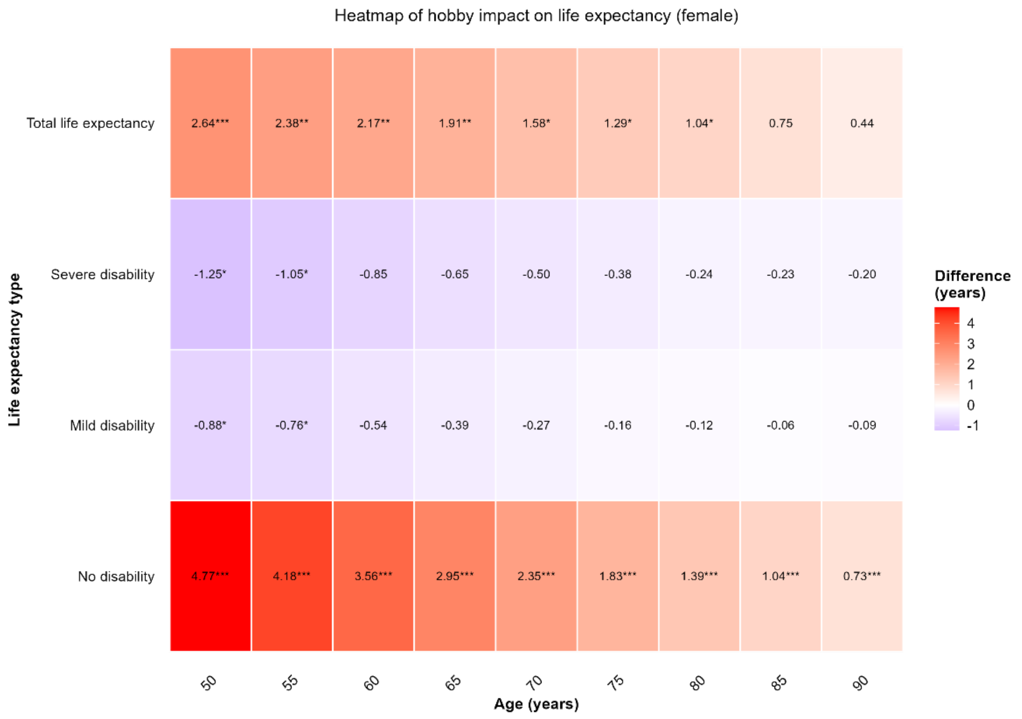

**Figure S10.** Heatmap of Life Expectancy Differences by Hobby

Participation Status Among Female Participants in the China Health and Retirement Longitudinal Study (CHARLS). Values represent differences in life expectancy (years). * indicate statistical significance levels: *** p<0.001, ** p<0.01, * p<0.05

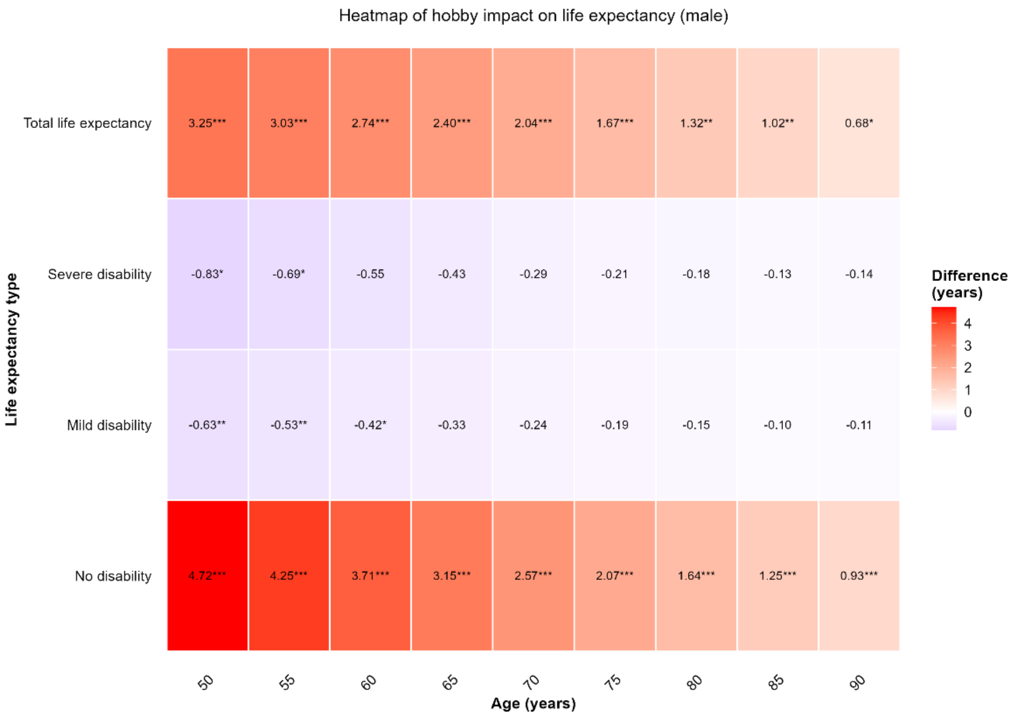


**Figure S11.** Heatmap of Life Expectancy Differences by Hobby

Participation Status Among Male Participants in the China Health and Retirement Longitudinal Study (CHARLS). Values represent differences in life expectancy (years). * indicate statistical significance levels: *** p<0.001, ** p<0.01, * p<0.05

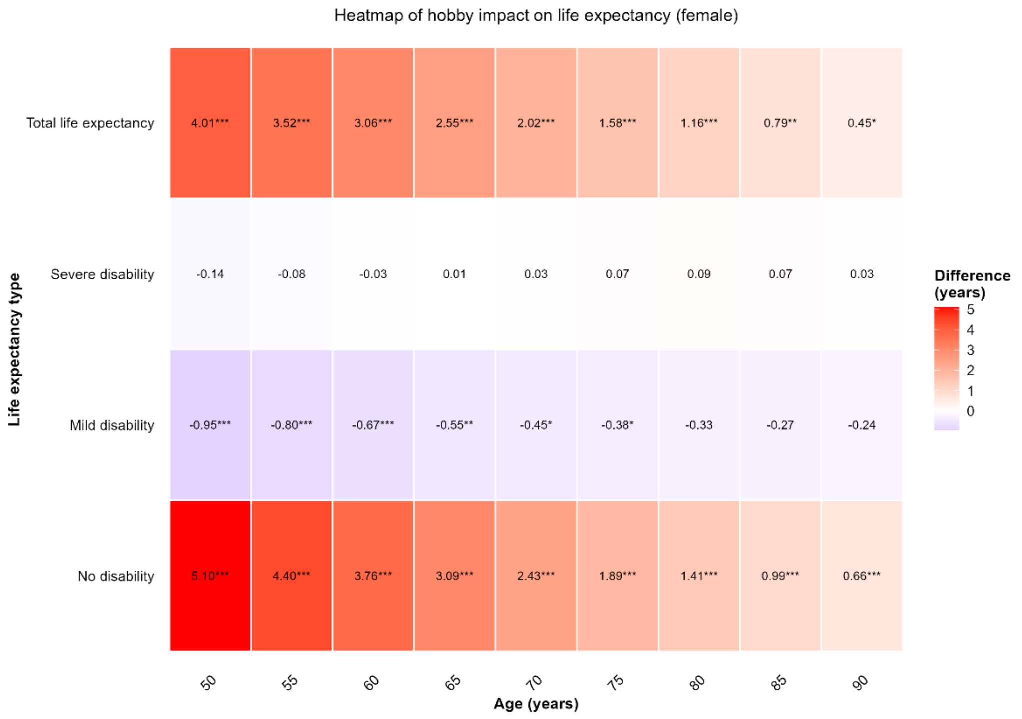


**Figure S12.** Heatmap of Life Expectancy Differences by Hobby Participation Status Among Female Participants in the Health and Retirement Study (HRS). Values represent differences in life expectancy (years). * indicate statistical significance levels: *** p<0.001, ** p<0.01, * p<0.05

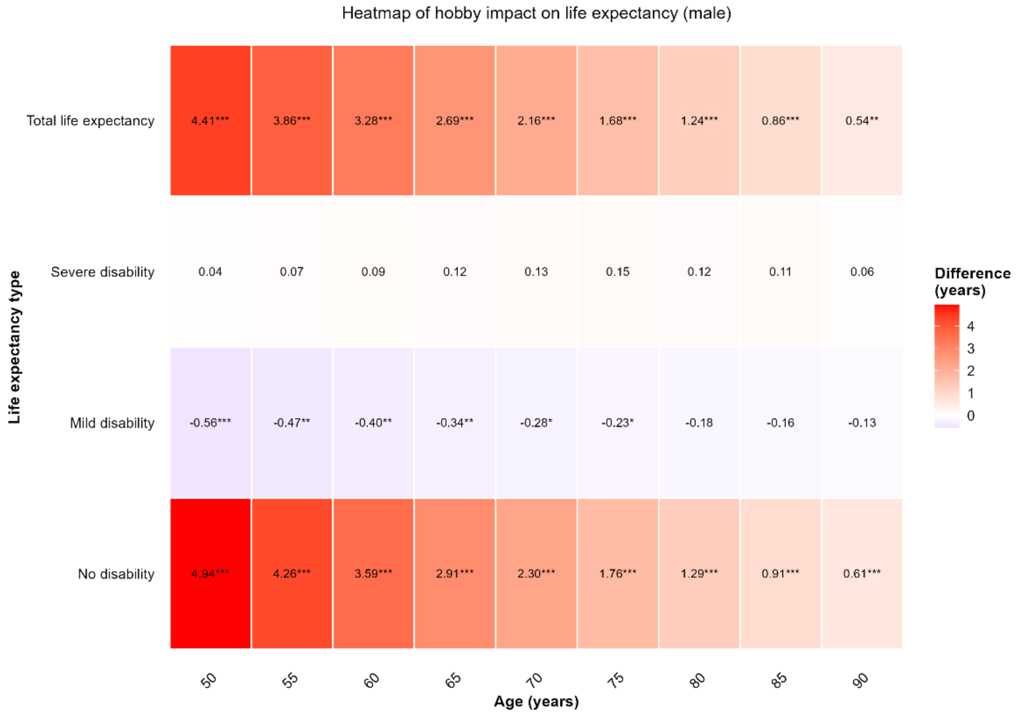

**Figure S13.** Heatmap of Life Expectancy Differences by Hobby

Participation Status Among Male Participants in the Health and Retirement Study (HRS). Values represent differences in life expectancy (years). * indicate statistical significance levels: *** p<0.001, ** p<0.01, * p<0.05

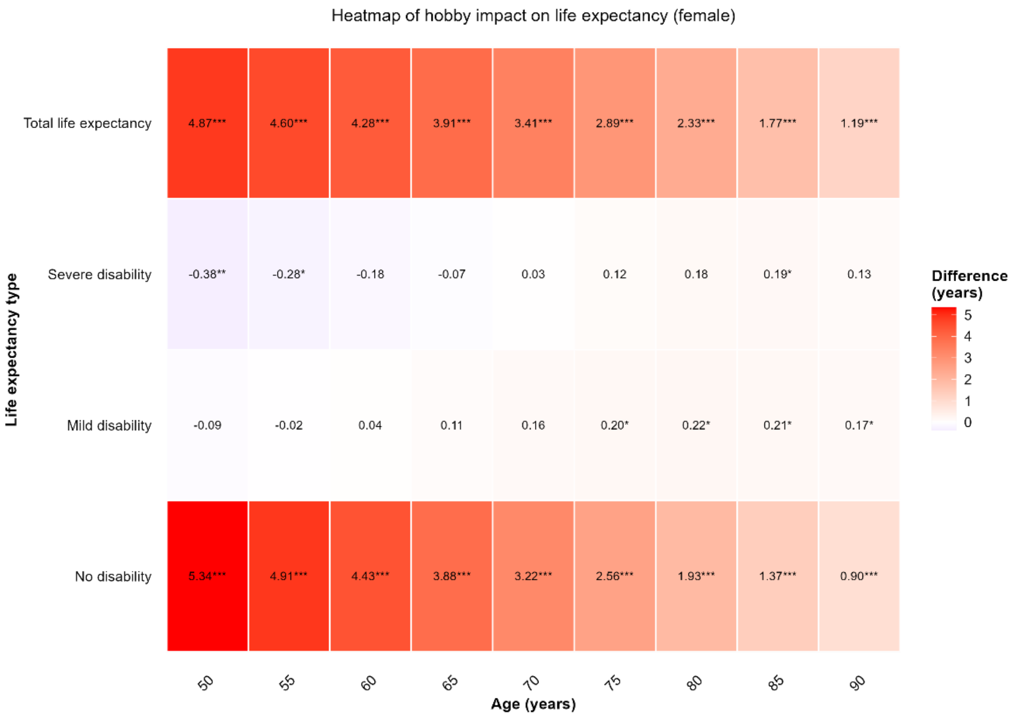


**Figure S14.** Heatmap of Life Expectancy Differences by Hobby

Participation Status Among Female Participants in the Survey of Health, Ageing and Retirement in Europe (SHARE). Values represent differences in life expectancy (years). * indicate statistical significance levels: *** p<0.001, ** p<0.01, * p<0.05

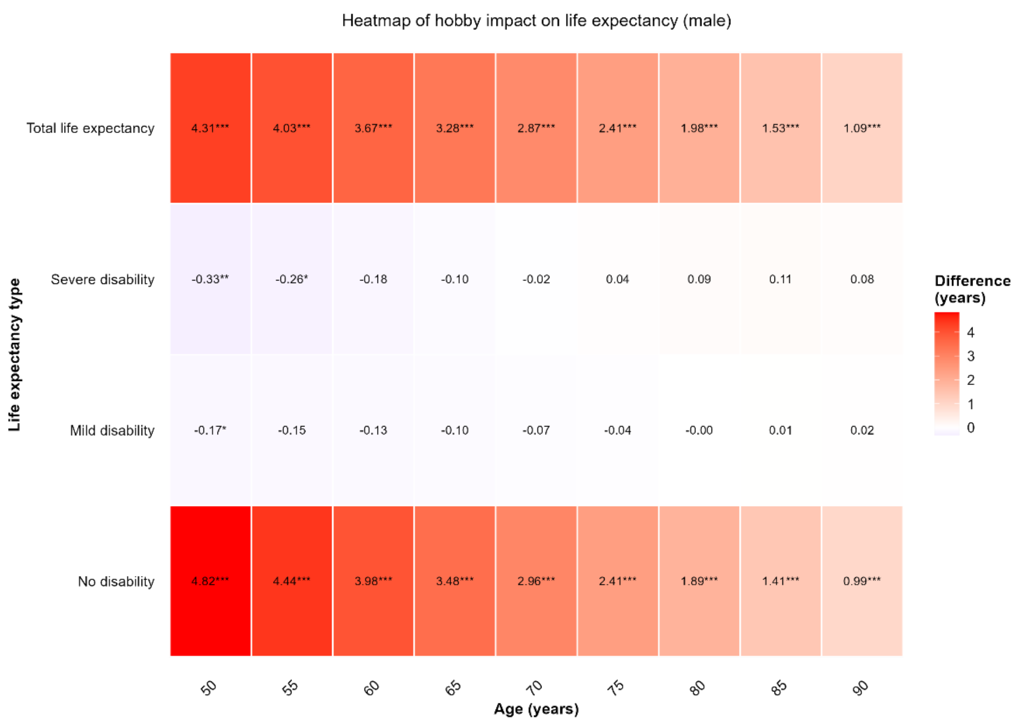


**Figure S15.** Heatmap of Life Expectancy Differences by Hobby

Participation Status Among Male Participants in the Survey of Health, Ageing and Retirement in Europe (SHARE). Values represent differences in life expectancy (years). * indicate statistical significance levels: *** p<0.001, ** p<0.01, * p<0.05

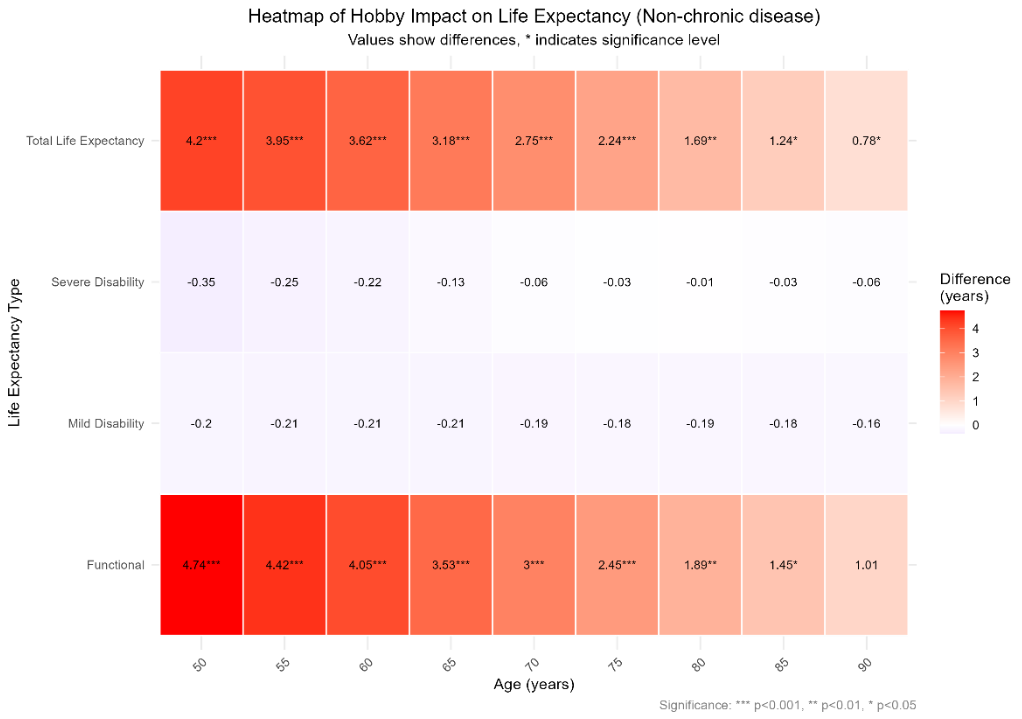

**Figure S16.** Heatmap of Life Expectancy Differences by Hobby

Participation Status Among Participants without Chronic Disease in the Mexican Health and Aging Study (MHAS). Values represent differences in life expectancy (years). * indicate statistical significance levels: *** p<0.001, ** p<0.01, * p<0.05

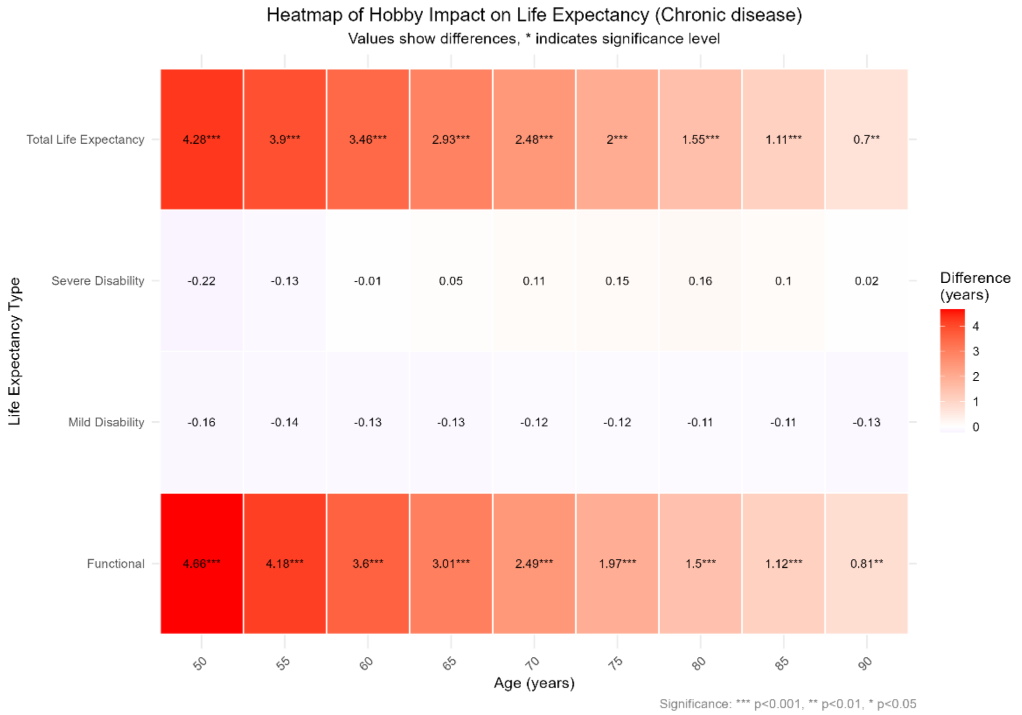


**Figure S17.** Heatmap of Life Expectancy Differences by Hobby

Participation Status Among Participants with Chronic Disease in the Mexican Health and Aging Study (MHAS). Values represent differences in life expectancy (years). * indicate statistical significance levels: *** p<0.001, ** p<0.01, * p<0.05

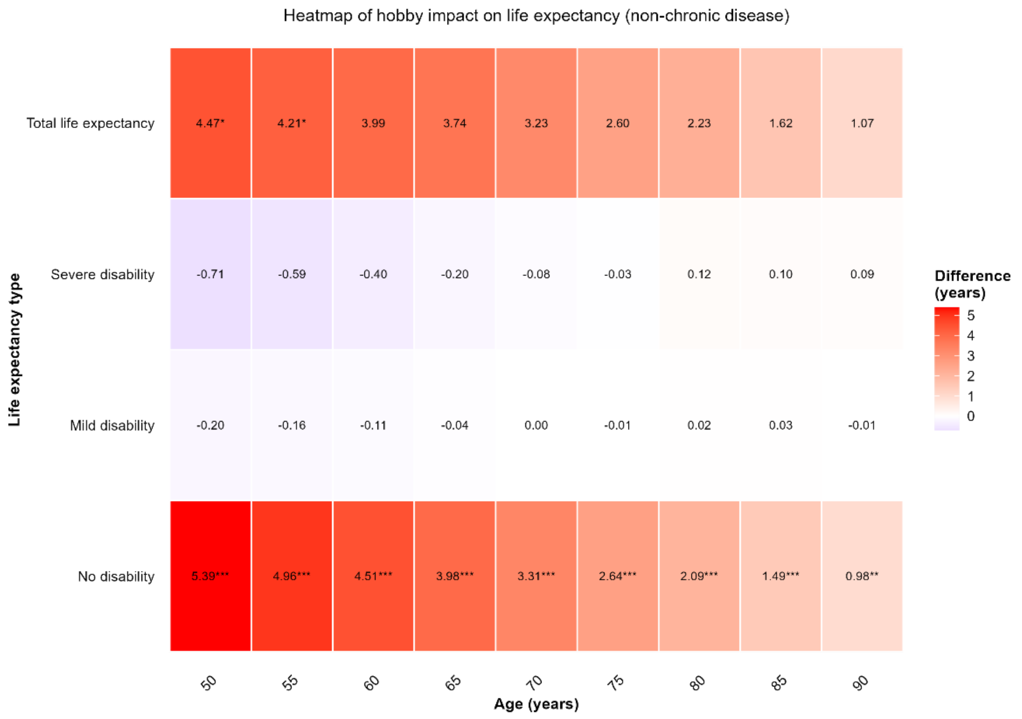


**Figure S18.** Heatmap of Life Expectancy Differences by Hobby

Participation Status Among Participants without Chronic Disease in the English Longitudinal Survey of Ageing (ELSA). Values represent differences in life expectancy (years). * indicate statistical significance levels: *** p<0.001, ** p<0.01, * p<0.05

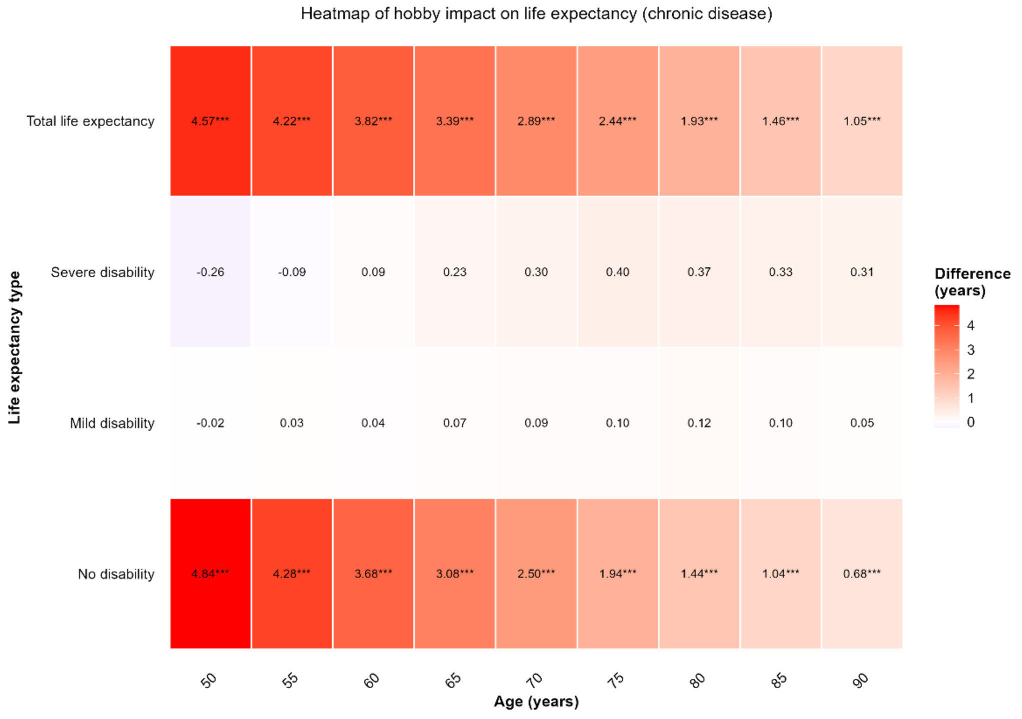

**Figure S19.** Heatmap of Life Expectancy Differences by Hobby

Participation Status Among Participants with Chronic Disease in the English Longitudinal Survey of Ageing (ELSA). Values represent differences in life expectancy (years). * indicate statistical significance levels: *** p<0.001, ** p<0.01, * p<0.05

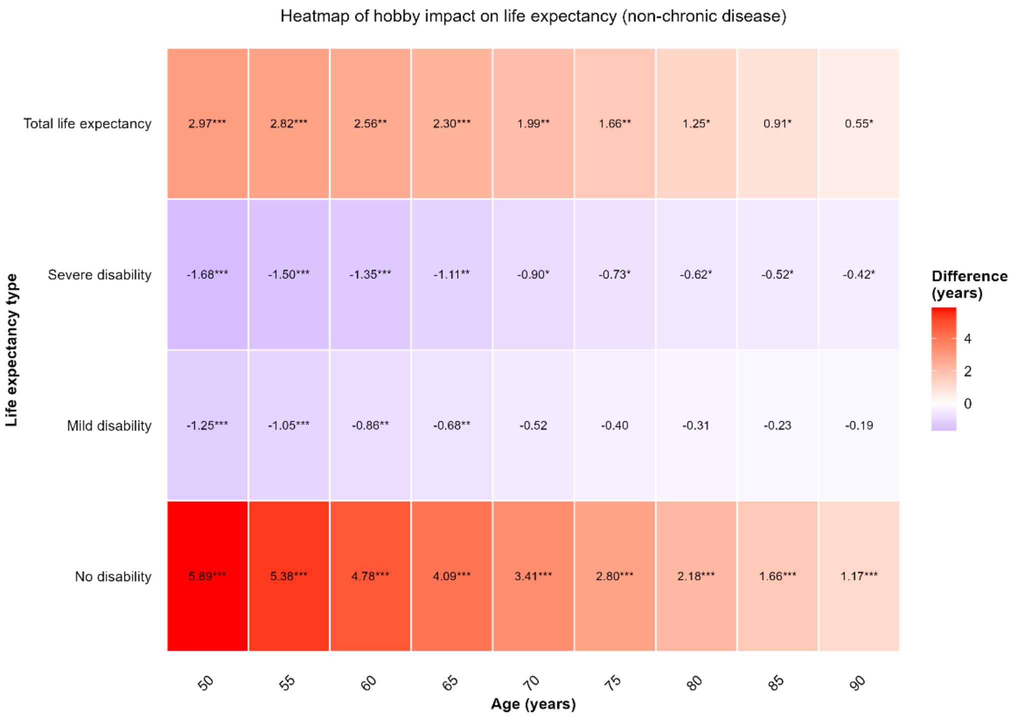


**Figure S20.** Heatmap of Life Expectancy Differences by Hobby

Participation Status Among Participants without Chronic Disease in the China Health and Retirement Longitudinal Study (CHARLS). Values represent differences in life expectancy (years). * indicate statistical significance levels: *** p<0.001, ** p<0.01, * p<0.05

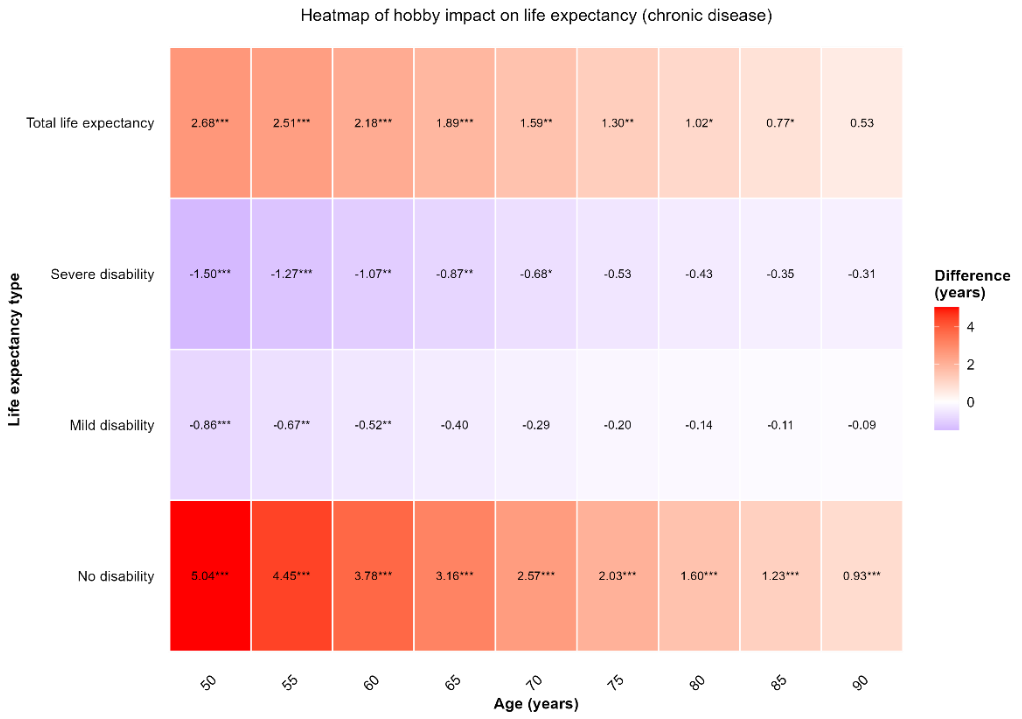


**Figure S21.** Heatmap of Life Expectancy Differences by Hobby Participation Status Among Participants with Chronic Disease in the China Health and Retirement Longitudinal Study (CHARLS). Values represent differences in life expectancy (years). * indicate statistical significance levels: *** p<0.001, ** p<0.01, * p<0.05

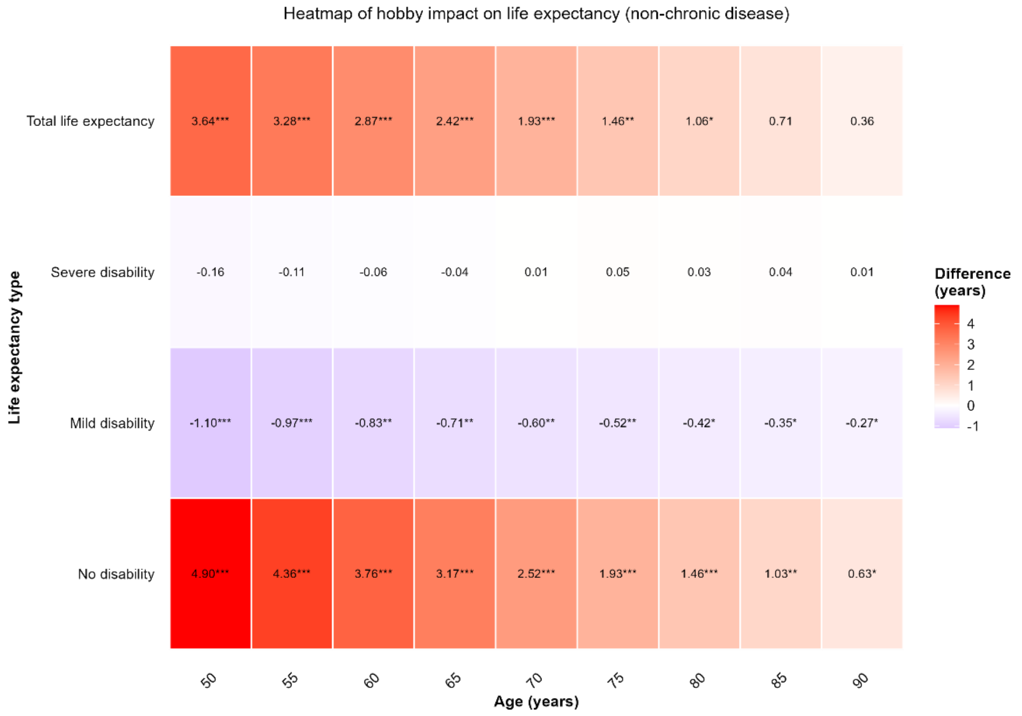

**Figure S22.** Heatmap of Life Expectancy Differences by Hobby

Participation Status Among Participants without Chronic Disease in the Health and Retirement Study (HRS). Values represent differences in life expectancy (years). * indicate statistical significance levels: *** p<0.001, ** p<0.01, * p<0.05

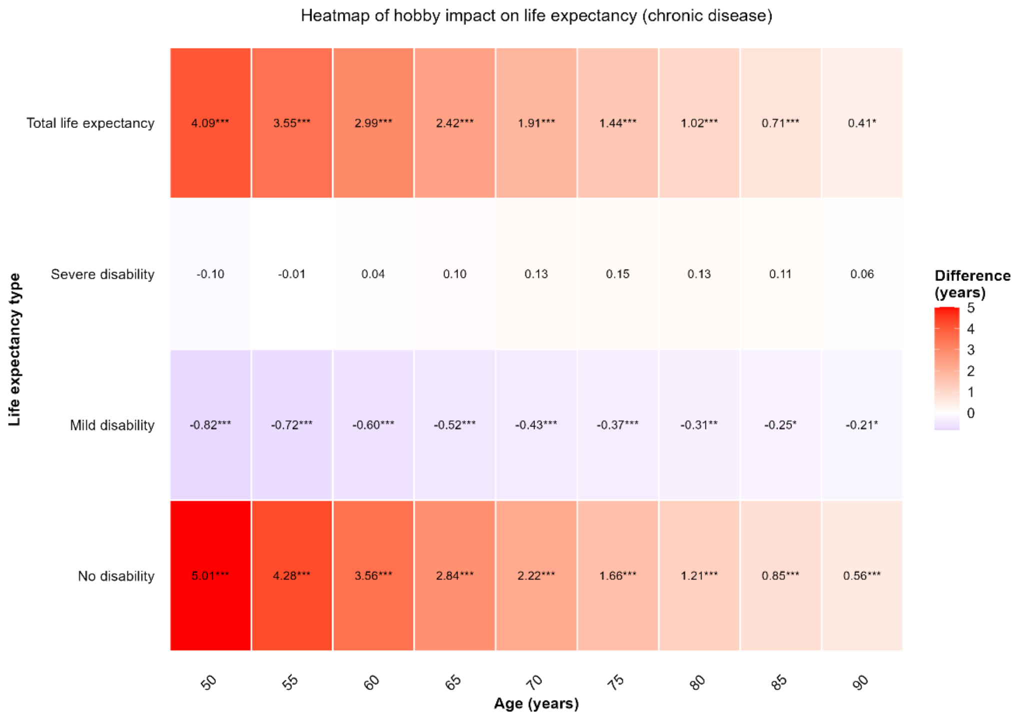


**Figure S23.** Heatmap of Life Expectancy Differences by Hobby

Participation Status Among Participants with Chronic Disease in the Health and Retirement Study (HRS). Values represent differences in life expectancy (years). * indicate statistical significance levels: *** p<0.001, ** p<0.01, * p<0.05

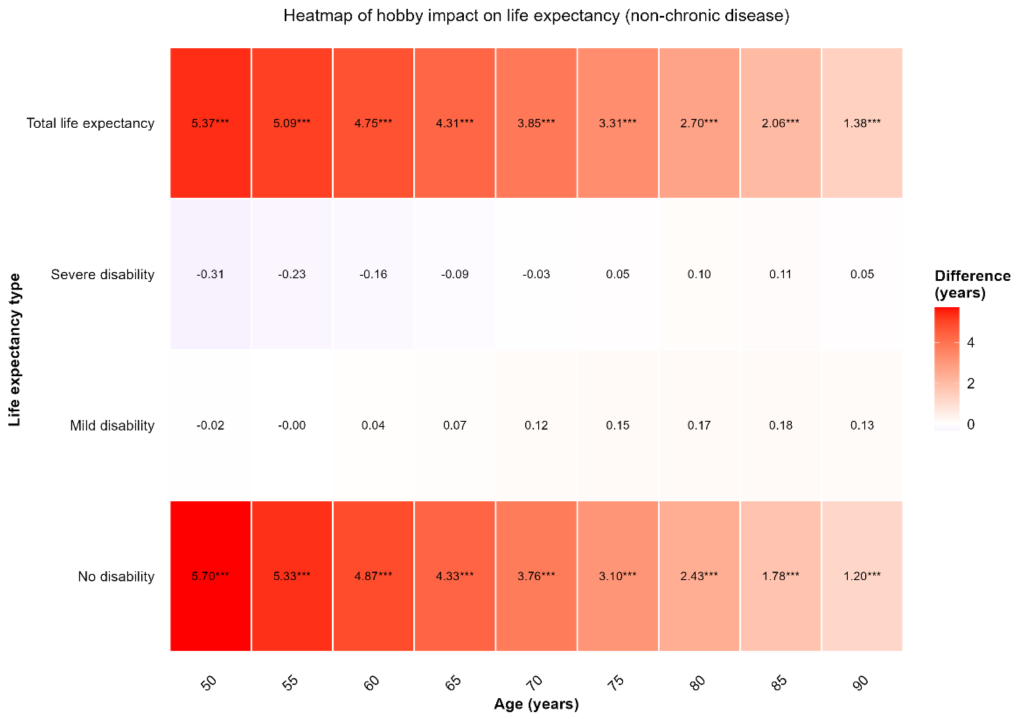


**Figure S24.** Heatmap of Life Expectancy Differences by Hobby

Participation Status Among Participants without Chronic Disease in the Survey of Health, Ageing and Retirement in Europe (SHARE). Values represent differences in life expectancy (years). * indicate statistical significance levels: *** p<0.001, ** p<0.01, * p<0.05

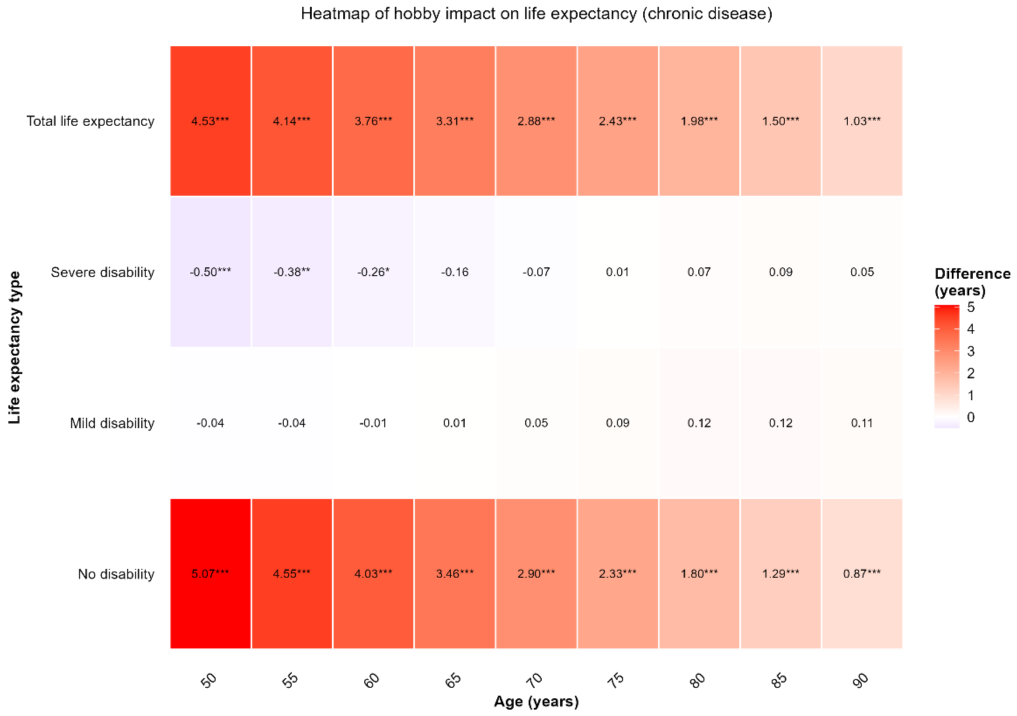

**Figure S25.** Heatmap of Life Expectancy Differences by Hobby

Participation Status Among Participants with Chronic Disease in the Survey of Health, Ageing and Retirement in Europe (SHARE). Values represent differences in life expectancy (years). * indicate statistical significance levels: *** p<0.001, ** p<0.01, * p<0.05

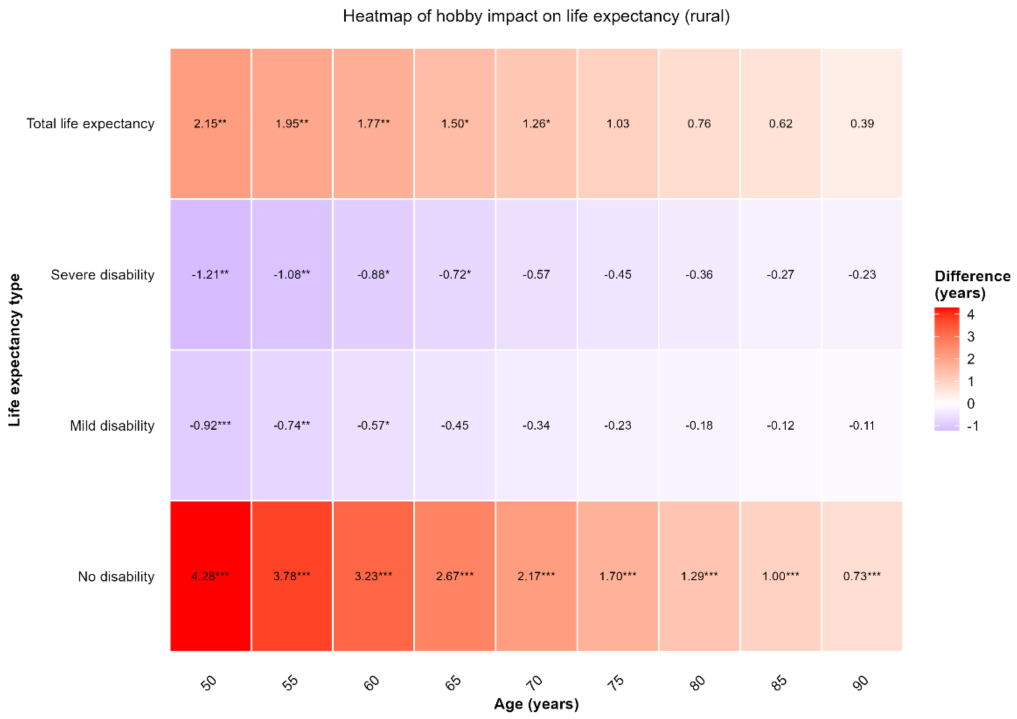


**Figure S26.** Heatmap of Life Expectancy Differences by Hobby

Participation Status Among Rural Participants in the China Health and Retirement Longitudinal Study (CHARLS). Values represent differences in life expectancy (years). * indicate statistical significance levels: *** p<0.001, ** p<0.01, * p<0.05

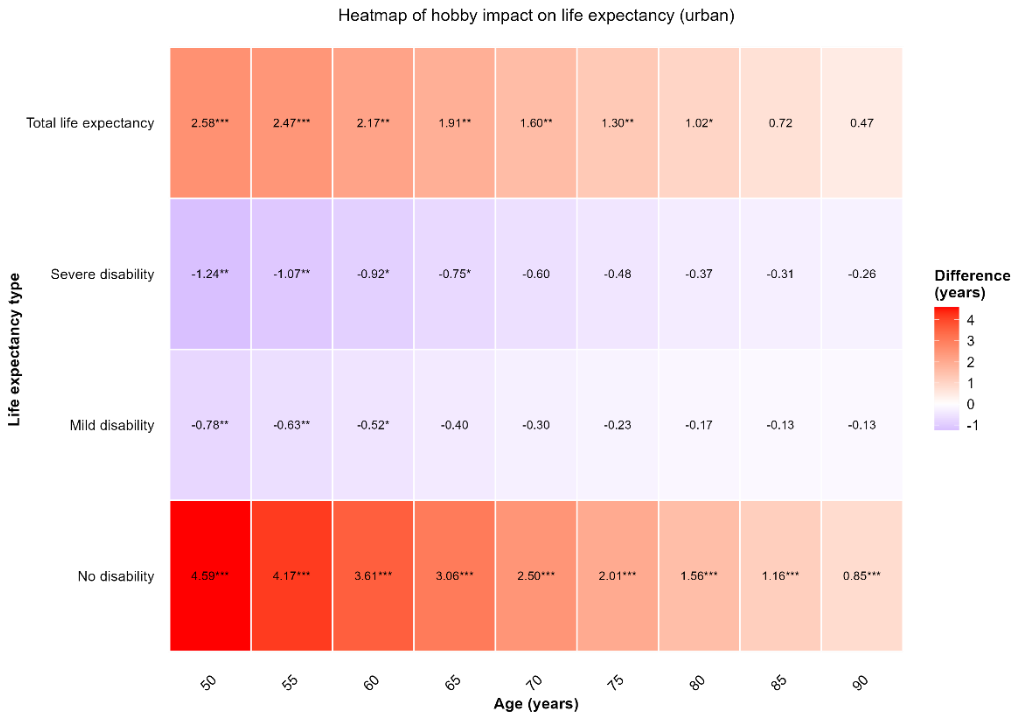


**Figure S27.** Heatmap of Life Expectancy Differences by Hobby Participation Status Among Urban Participants in the China Health and Retirement Longitudinal Study (CHARLS). Values represent differences in life expectancy (years). * indicate statistical significance levels: *** p<0.001, ** p<0.01, * p<0.05

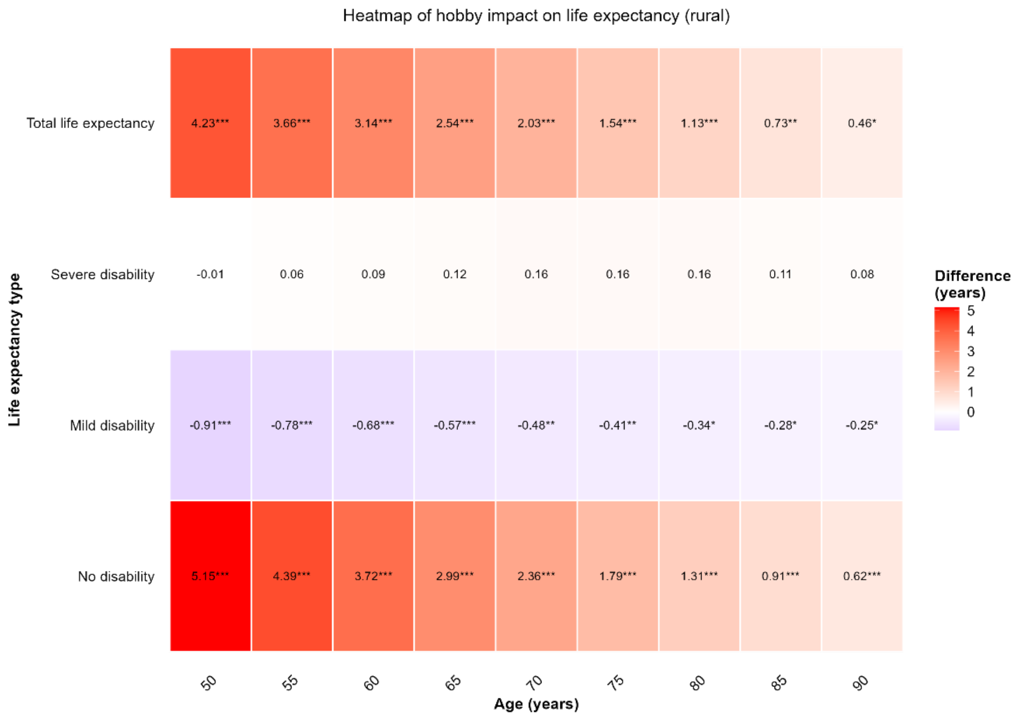

**Figure S28.** Heatmap of Life Expectancy Differences by Hobby Participation Status Among Rural Participants in the Health and Retirement Study (HRS). Values represent differences in life expectancy (years). * indicate statistical significance levels: *** p<0.001, ** p<0.01, * p<0.05

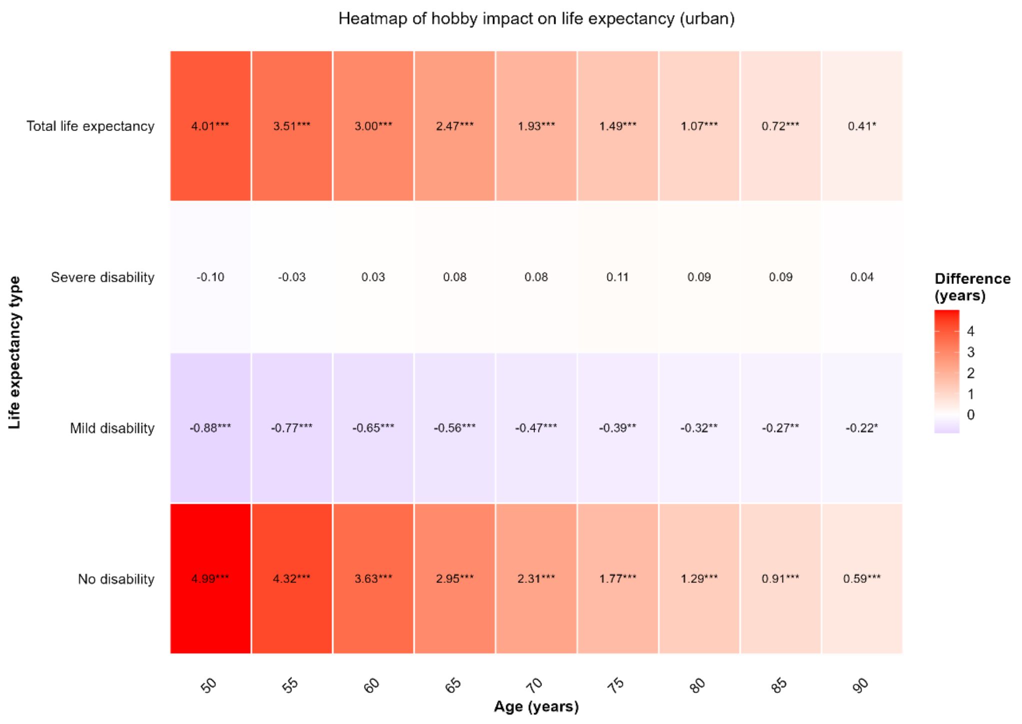


**Figure S29.** Heatmap of Life Expectancy Differences by Hobby Participation Status Among Urban Participants in the Health and Retirement Study (HRS). Values represent differences in life expectancy (years). * indicate statistical significance levels: *** p<0.001, ** p<0.01, * p<0.05

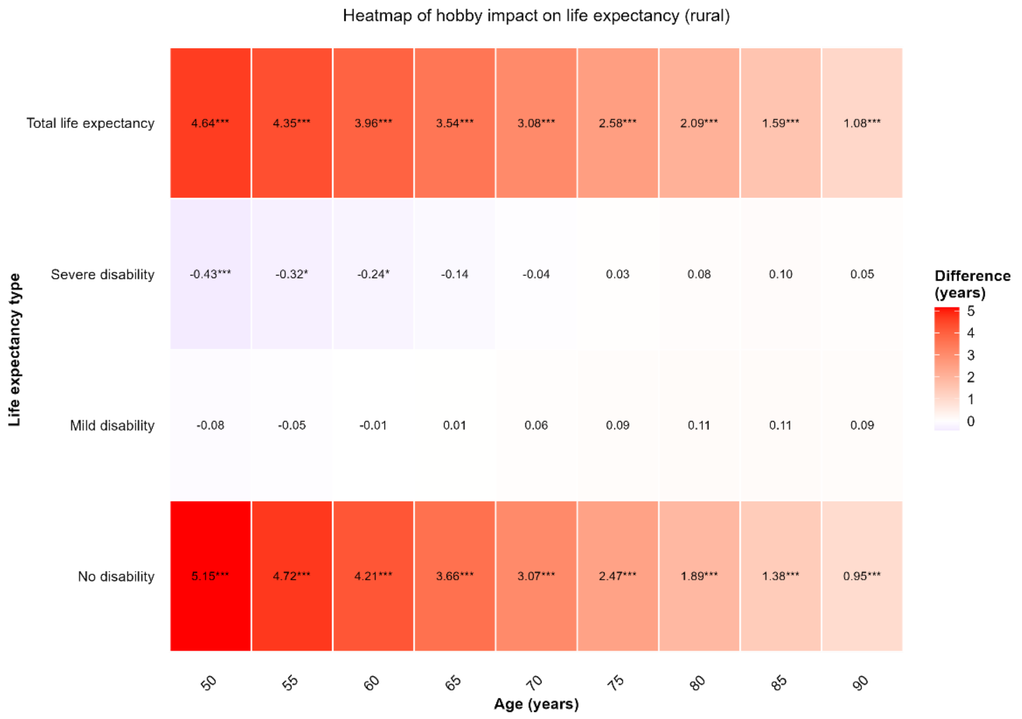


**Figure S30.** Heatmap of Life Expectancy Differences by Hobby

Participation Status Among Rural Participants in the Survey of Health, Ageing and Retirement in Europe (SHARE). Values represent differences in life expectancy (years). * indicate statistical significance levels: *** p<0.001, ** p<0.01, * p<0.05

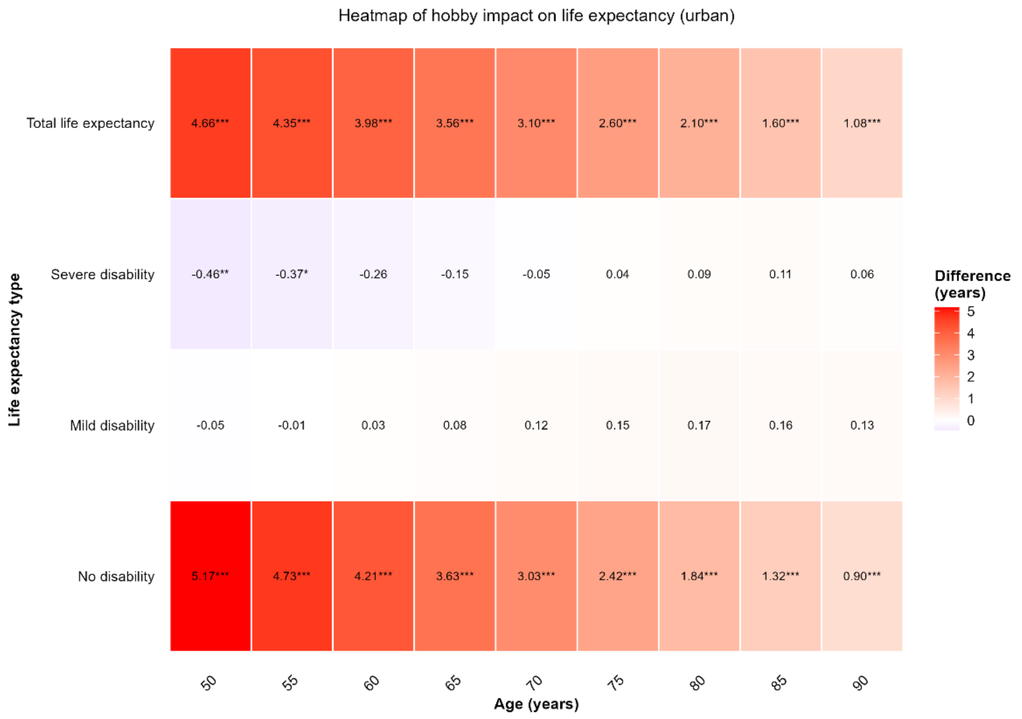

**Figure S31.** Heatmap of Life Expectancy Differences by Hobby

Participation Status Among Urban Participants in the Survey of Health, Ageing and Retirement in Europe (SHARE). Values represent differences in life expectancy (years). * indicate statistical significance levels: *** p<0.001, ** p<0.01, * p<0.05

**Table S7.** Associations between Hobby Participation and Disability Transitions by Cohort (Further Adjusted)

| State Transition | CHARLS HR(95%CI) | HRS HR(95%CI) | SHARE HR(95%CI) |
|---|---|---|---|
| State 1 - State 2 | 0.80* (0.66,0.97) | 0.65* (0.54,0.78) | 0.69* (0.61,0.77) |
| State 1 - State 3 | 0.98 (0.82,1.18) | 0.98 (0.82,1.16) | 0.98 (0.82,1.19) |
| State 1 - State 4 | \ | 0.59* (0.40,0.85) | 1.17 (0.83,1.63) |
| State 2 - State 1 | 1.11 (0.90,1.37) | 0.91 (0.73,1.15) | 1.06 (0.91,1.23) |
| State 2 - State 3 | 0.89 (0.65,1.22) | 0.81 (0.61,1.08) | 0.95 (0.80,1.12) |
| State 2 - State 4 | 1.39 (0.95,2.04) | 1.11 (0.81,1.53) | 1.12 (0.84,1.50) |
| State 3 - State 1 | 1.47* (1.19,1.80) | 1.75* (1.36,2.26) | 1.22* (1.01,1.47) |
| State 3 - State 2 | 0.97 (0.70,1.33) | 1.34 (0.94,1.90) | 1.38* (1.05,1.83) |
| State 3 - State 4 | 0.70 (0.47,1.03) | 0.86* (0.76,0.97) | 0.76* (0.69,0.85) |

Note: *CHARLS – China Health and Retirement Longitudinal Study; HRS – Health and Retirement Study; SHARE – Survey of Health, Ageing and Retirement in Europe; HR – Hazard ratios; CI – Confidence Interval.

†State 1 = functional independence; State 2 = mild disability; State 3 = severe disability; State 4 = death; * indicates 5% significant ratio; \ indicates that this transition has not been incorporated into the multi-state model, as it is rarely observed.

**Table S8.** Associations between Hobby Participation and Disability Transitions by Cohort (Three-State Model)

| State Transition | MHAS HR(95%CI) | ELSA HR(95%CI) | CHARLS HR(95%CI) | HRS HR(95%CI) | SHARE HR(95%CI) |
|---|---|---|---|---|---|
| State 1 - State 2 | 0.81* (0.74,0.88) | 0.81* (0.74,0.88) | 0.79* (0.73,0.85) | 0.77* (0.72,0.82) | 0.76* (0.72,0.80) |
| State 1 - State 3 | \ | 1.41 (0.44,4.46) | 0.77 (0.31,1.93) | 0.91 (0.62,1.35) | 1.65 (1.06,2.57) |
| State 2 - State 1 | 1.31* (1.17,1.48) | 1.27*(1.14,1.41) | 1.27* (1.16,1.40) | 1.31* (1.17,1.47) | 1.40* (1.31,1.49) |
| State 2 - State 3 | 0.84* (0.76,0.92) | 0.81* (0.73,0.90) | 0.86* (0.75,1.00) | 0.86* (0.81,0.92) | 0.74* (0.71,0.78) |

Note: *MHAS – Mexican Health and Aging Study; ELSA – English Longitudinal Study of Ageing; CHARLS – China Health and Retirement Longitudinal Study; HRS – Health and Retirement Study; SHARE – Survey of Health, Ageing and Retirement in Europe; HR – Hazard ratios; CI – Confidence Interval.

†State 1 = functional independence; State 2 = disability; State 3 = death; * indicates 5% significant ratio; \ indicates that this transition has not been incorporated into the multi-state model, as it is rarely observed.

‡All cohorts were adjusted for age, gender, live alone, marital status, education level, smoke, drink, and chronic disease. HRS and SHARE further adjusted for household income level, while MHAS and ELSA additionally adjusted for household income level and cognitive function.

**Table S9.** Associations between Hobby Participation and Disability Transitions by Cohort (ADL-Based Definition)

| State Transition | MHAS HR(95%CI) | ELSA HR(95%CI) | CHARLS HR(95%CI) | HRS HR(95%CI) | SHARE HR(95%CI) |
|---|---|---|---|---|---|
| State 1 - State 2 | 0.83 (0.65,1.05) | 0.86* (0.75,0.98) | 0.85* (0.72,0.99) | 0.87* (0.76,0.99) | 0.85* (0.77,0.93) |
| State 1 - State 3 | 0.58* (0.38,0.89) | 0.44*(0.28,0.68) | 0.52* (0.35,0.77) | 1.00 (0.71,1.41) | 0.50* (0.42,0.58) |
| State 1 - State 4 | \ | 1.40 (0.61,3.20) | \ | 0.70* (0.62,0.80) | 0.88 (0.77,1.01) |
| State 2 - State 1 | 1.03 (0.85,1.25) | 1.08 (0.93,1.25) | 0.95 (0.80,1.13) | 1.12 (0.95,1.32) | 1.16 (0.99,1.36) |
| State 2 - State 3 | 1.08 (0.79,1.47) | 0.84 (0.68,1.05) | 0.85 (0.64,1.12) | 0.80* (0.69,0.93) | 0.74* (0.65,0.84) |
| State 2 - State 4 | 1.09 (0.31,3.84) | 0.79* (0.65,0.97) | 1.12 (0.77,1.63) | \ | \ |
| State 3 - State 1 | 1.39 (0.70,2.75) | 1.13 (0.56,2.27) | 1.23 (0.73,2.10) | 1.41 (0.70,2.83) | 0.81 (0.52,1.27) |
| State 3 - State 2 | 1.45* (1.09,1.93) | 1.25* (1.02,1.52) | 1.44* (1.04,2.02) | 1.38* (1.05,1.81) | 1.84* (1.41,2.42) |
| State 3 - State 4 | 0.87* (0.77,0.99) | 0.50* (0.32,0.80) | 0.95 (0.74,1.22) | 0.95 (0.85,1.06) | 0.91* (0.85,0.97) |

Note: *ADL – activities of daily living; MHAS – Mexican Health and Aging Study; ELSA – English Longitudinal Study of Ageing; CHARLS – China Health and Retirement Longitudinal Study; HRS – Health and Retirement Study; SHARE – Survey of Health, Ageing and Retirement in Europe; HR – Hazard ratios; CI – Confidence Interval.

†State 1 = functional independence; State 2 = mild disability; State 3 = severe disability; State 4 = death. * indicates 5% significant ratio; \ indicates that this transition has not been incorporated into the multi-state model, as it is rarely observed.

‡All cohorts were adjusted for age, gender, live alone, marital status, education level, smoke, drink, and chronic disease. HRS and SHARE further adjusted for household

income level, while MHAS and ELSA additionally adjusted for household income level and cognitive function.

**Table S10.** Associations between Hobby Participation and Disability Transitions by Cohort (Allowing Missing Follow-up Covariates)

| State Transition | MHAS HR(95%CI) | ELSA HR(95%CI) | CHARLS HR(95%CI) | HRS HR(95%CI) | SHARE HR(95%CI) |
|---|---|---|---|---|---|
| State 1 - State 2 | 0.81 (0.59,1.11) | 0.82* (0.70,0.97) | 0.68* (0.57,0.80) | 0.75* (0.66,0.86) | 0.68* (0.63,0.73) |
| State 1 - State 3 | 0.79* (0.66,0.95) | 0.83* (0.70,0.98) | 0.92 (0.8,1.07) | 0.85* (0.76,0.94) | 1.05 (0.92,1.20) |
| State 1 - State 4 | \ | \ | \ | 0.82 (0.59,1.14) | 1.07 (0.74,1.54) |
| State 2 - State 1 | 1.10 (0.74,1.65) | 0.96 (0.79,1.16) | 1.09 (0.91,1.3) | 1.09 (0.95,1.26) | 1.16 (1.05,1.28) |
| State 2 - State 3 | 1.03 (0.72,1.46) | 0.95 (0.75,1.19) | 0.82 (0.63,1.05) | 0.92 (0.76,1.11) | 0.88* (0.79,0.98) |
| State 2 - State 4 | 1.32 (0.67,2.61) | 1.10 (0.79,1.53) | \ | 1.06 (0.76,1.48) | 1.30 (0.83,2.04) |
| State 3 - State 1 | 1.38* (1.18,1.61) | 1.70* (1.37,2.10) | 1.59* (1.33,1.91) | 1.44* (1.22,1.69) | 1.46* (1.29,1.65) |
| State 3 - State 2 | 0.79 (0.47,1.33) | 0.86 (0.68,1.09) | 0.90 (0.67,1.19) | 1.12 (0.89,1.40) | 1.62* (1.37,1.91) |
| State 3 - State 4 | 0.78* (0.65,0.93) | 0.71* (0.59,0.87) | 0.91 (0.79,1.04) | 0.83* (0.75,0.91) | 0.73* (0.67,0.79) |

Note: *ADL – activities of daily living; MHAS – Mexican Health and Aging Study; ELSA – English Longitudinal Study of Ageing; CHARLS – China Health and Retirement Longitudinal Study; HRS – Health and Retirement Study; SHARE – Survey of Health, Ageing and Retirement in Europe; HR – Hazard ratios; CI – Confidence Interval.

†State 1 = functional independence; State 2 = mild disability; State 3 = severe disability; State 4 = death. * indicates 5% significant ratio; \ indicates that this

transition has not been incorporated into the multi-state model, as it is rarely observed.
‡All cohorts were adjusted for age, gender, live alone, marital status, education level, smoke, drink, and chronic disease. HRS and SHARE further adjusted for household income level, while MHAS and ELSA additionally adjusted for household income level and cognitive function.

**Table S11.** Comparison summary of baseline characteristics between the included participants and excluded participants in MHAS

| Characteristic | | Included participants (n=13850) | Excluded participants (n=9512) | P-value |
|---|---|---|---|---|
| Age group | 50-59 | 4532 (32.7%) | 4715 (49.6%) | <0.001 |
| | 60-69 | 5180 (37.4%) | 1427 (15.0%) | |
| | >=70 | 4138 (29.9%) | 3370 (35.4%) | |
| Gender | Female | 7929 (57.2%) | 4913 (51.7%) | <0.001 |
| | Male | 5921 (42.8%) | 4599 (48.3%) | |
| Live alone | No | 13017 (94%) | 5957 (92.7%) | <0.001 |
| | Yes | 833 (6.0%) | 471 (7.3%) | |
| Marital status | No | 4245 (30.6%) | 1674 (26.0%) | <0.001 |
| | Yes | 9605 (69.4%) | 4754 (74.0%) | |
| Education level | Less than upper secondary education | 11980 (86.5%) | 7464 (81.1%) | <0.001 |
| | Upper secondary and vocational training | 468 (3.4%) | 216 (2.3%) | |
| | Tertiary education | 1402 (10.1%) | 1521 (16.5%) | |
| Household income level | Low | 4960 (35.8%) | 2185 (35.2%) | <0.001 |
| | Middle | 4237 (30.6%) | 1651 (26.6%) | |
| | High | 4653 (33.6%) | 2375 (38.2%) | |

| | | | | |
|---|---|---|---|---|
| Smoke | No | 8651 (62.5%) | 3901 (60.8%) | 0.021 |
| | Yes | 5199 (37.5%) | 2518 (39.2%) | |
| Drink | No | 10632 (76.8%) | 4432 (69.0%) | <0.001 |
| | Yes | 3218 (23.2%) | 1993 (31.0%) | |
| Cognitive impairment | No | 11612 (83.8%) | 4459 (84.7%) | 0.159 |
| | Yes | 2238 (16.2%) | 807 (15.3%) | |
| Chronic disease | No | 5038 (36.4%) | 3095 (36.4%) | 0.972 |
| | Yes | 8812 (63.6%) | 5408 (63.6%) | |

Note: *MHAS – Mexican Health and Aging Study.
†Number (percentage [%]) was used to describe categorical variables and the Pearson chi-square test for categorical variables.

**Table S12.** Comparison summary of baseline characteristics between the included participants and excluded participants in ELSA

| Characteristic | | Included participants (n=11794) | Excluded participants (n=5865) | P-value |
|---|---|---|---|---|
| Age group | 50-59 | 5243 (44.5%) | 3327 (56.7%) | <0.001 |
| | 60-69 | 3386 (28.7%) | 1256 (21.4%) | |
| | >=70 | 3165 (26.8%) | 1282 (21.9%) | |
| Gender | Female | 6226 (52.8%) | 3354 (57.2%) | <0.001 |
| | Male | 5568 (47.2%) | 2511 (42.8%) | |
| Live alone | No | 9219 (78.2%) | 4610 (81.8%) | <0.001 |
| | Yes | 2575 (21.8%) | 1023 (18.2%) | |
| Marital status | No | 3190 (27.0%) | 1487 (26.4%) | 0.383 |
| | Yes | 8604 (73.0%) | 4141 (73.6%) | |

| | | | | |
|---|---|---|---|---|
| Education level | Less than upper secondary education | 5108 (43.3%) | 1775 (40.4%) | <0.001 |
| | Upper secondary and vocational training | 2298 (19.5%) | 1947 (44.3%) | |
| | Tertiary education | 4388 (37.2%) | 669 (15.2%) | |
| Household income level | Low | 3511 (29.8%) | 1806 (33.4%) | <0.001 |
| | Middle | 3868 (32.8%) | 1748 (32.4%) | |
| | High | 4415 (37.4%) | 1846 (34.2%) | |
| Smoke | No | 4400 (37.3%) | 2085 (40.0%) | 0.001 |
| | Yes | 7394 (62.7%) | 3132 (60.0%) | |
| Drink | No | 1233 (10.5%) | 604 (13.5%) | <0.001 |
| | Yes | 10561 (89.5%) | 3880 (86.5%) | |
| Cognitive impairment | No | 10089 (85.5%) | 3989 (80.2%) | <0.001 |
| | Yes | 1705 (14.5%) | 982 (19.8%) | |
| Chronic disease | No | 6033 (51.2%) | 2928 (51.4%) | 0.764 |
| | Yes | 5761 (48.8%) | 2769 (48.6%) | |

Note: *ELSA – English Longitudinal Study of Ageing.
†Number (percentage [%]) was used to describe categorical variables and the Pearson chi-square test for categorical variables.

**Table S13.** Comparison summary of baseline characteristics between the included participants and excluded participants in CHARLS

| Characteristic | | Included participants (n=16261) | Excluded participants (n=6125) | P-value |
|---|---|---|---|---|
| Age group | 50-59 | 8639 (53.1%) | 4630 (75.6%) | <0.001 |
| | 60-69 | 4897 (30.1%) | 857 (14.0%) | |

| | | | | |
|---|---|---|---|---|
| | >=70 | 2725 (16.8%) | 638 (10.4%) | |
| Gender | Female | 8293 (51.0%) | 3035 (49.6%) | 0.053 |
| | Male | 7968 (49.0%) | 3090 (50.4%) | |
| Residence | Rural | 9840 (60.5%) | 2495 (44.1%) | <0.001 |
| | Urban | 6421 (39.5%) | 3164 (55.9%) | |
| Live alone | No | 15304 (94.1%) | 4891 (92.9%) | 0.001 |
| | Yes | 957 (5.9%) | 375 (7.1%) | |
| Marital status | No | 2156 (13.3%) | 611 (11.6%) | 0.002 |
| | Yes | 14105 (86.7%) | 4647 (88.4%) | |
| Education level | Less than upper secondary education | 14268 (87.7%) | 5089 (83.3%) | <0.001 |
| | Upper secondary and vocational training | 1274 (7.8%) | 760 (12.4%) | |
| | Tertiary education | 719 (4.4%) | 262 (4.3%) | |
| Smoke | No | 9533 (58.6%) | 2965 (58.6%) | 0.937 |
| | Yes | 6728 (41.4%) | 2098 (41.4%) | |
| Drink | No | 9489 (58.4%) | 2609 (51.6%) | <0.001 |
| | Yes | 6772 (41.6%) | 2446 (48.4%) | |
| Chronic disease | No | 8909 (54.8%) | 2076 (53.1%) | 0.056 |
| | Yes | 7352 (45.2%) | 1834 (46.9%) | |

Note: *CHARLS – China Health and Retirement Longitudinal Study.
†Number (percentage [%]) was used to describe categorical variables and the Pearson chi-square test for categorical variables.

**Table S14.** Comparison summary of baseline characteristics between the included participants and excluded participants in HRS

| Characteristic | | Included participants (n=15938) | Excluded participants (n=15095) | P-value |
|---|---|---|---|---|
| Age group | 50-59 | 4950 (31.1%) | 8547 (56.6%) | <0.001 |
| | 60-69 | 4439 (27.9%) | 2310 (15.3%) | |
| | >=70 | 6549 (41.1%) | 4238 (28.1%) | |
| Gender | Female | 9237 (58.0%) | 8442 (55.9%) | <0.001 |
| | Male | 6701 (42.0%) | 6653 (44.1%) | |
| Residence | Rural | 4845 (30.4%) | 3384 (23.3%) | <0.001 |
| | Urban | 11093 (69.6%) | 11141 (76.7%) | |
| Live alone | No | 12397 (77.8%) | 10491 (79.1%) | 0.008 |
| | Yes | 3541 (22.2%) | 2777 (20.9%) | |
| Marital status | No | 5632 (35.3%) | 5041 (38.0%) | <0.001 |
| | Yes | 10306 (64.7%) | 8219 (62.0%) | |
| Education level | Less than upper secondary education | 2781 (17.4%) | 3641 (24.1%) | <0.001 |
| | Upper secondary and vocational training | 5556 (34.9%) | 8465 (56.1%) | |
| | Tertiary education | 7601 (47.7%) | 2982 (19.8%) | |
| Household income level | Low | 4946 (31.0%) | 4700 (35.4%) | <0.001 |
| | Middle | 5376 (33.7%) | 3901 (29.4%) | |
| | High | 5616 (35.2%) | 4671 (35.2%) | |
| Smoke | No | 6886 (43.2%) | 5891 (44.9%) | 0.003 |
| | Yes | 9052 (56.8%) | 7225 (55.1%) | |
| Drink | No | 7215 (45.3%) | 5435 (41.0%) | <0.001 |
| | Yes | 8723 (54.7%) | 7830 (59.0%) | |
| | No | 4312 (27.1%) | 4470 (30.2%) | <0.001 |

| | | | | |
|---|---|---|---|---|
| Chronic disease | Yes | 11626 (72.9%) | 10320 (69.8%) | |

Note: *HRS – Health and Retirement Study.
†Number (percentage [%]) was used to describe categorical variables and the Pearson chi-square test for categorical variables.

**Table S15.** Comparison summary of baseline characteristics between the included participants and excluded participants in SHARE

| Characteristic | | Included participants (n=69807) | Excluded participants (n=57675) | P-value |
|---|---|---|---|---|
| Age group | 50-59 | 21612 (31.0%) | 21582 (37.4%) | <0.001 |
| | 60-69 | 24215 (34.7%) | 17859 (31.0%) | |
| | >=70 | 23980 (34.4%) | 18234 (31.6%) | |
| Gender | Female | 38651 (55.4%) | 32042 (55.6%) | 0.502 |
| | Male | 31156 (44.6%) | 25633 (44.4%) | |
| Residence | Rural | 47232 (67.7%) | 32491 (64.1%) | <0.001 |
| | Urban | 22575 (32.3%) | 18183 (35.9%) | |
| Live alone | No | 55826 (80.0%) | 44795 (80.3%) | 0.128 |
| | Yes | 13981 (20.0%) | 10978 (19.7%) | |
| Marital status | No | 17011 (24.4%) | 14166 (25.5%) | <0.001 |
| | Yes | 52796 (75.6%) | 41424 (74.5%) | |
| Education level | Less than upper secondary education | 28959 (41.5%) | 20957 (36.3%) | <0.001 |
| | Upper secondary and vocational training | 25926 (37.1%) | 24505 (42.5%) | |
| | Tertiary education | 14922 (21.4%) | 12213 (21.2%) | |
| | Low | 22519 (32.3%) | 13005 (35.3%) | <0.001 |

| | | | | |
|---|---|---|---|---|
| Household income level | Middle | 23352 (33.5%) | 11970 (32.5%) | |
| | High | 23936 (34.3%) | 11831 (32.1%) | |
| Smoke | No | 37018 (53.0%) | 19643 (54.7%) | <0.001 |
| | Yes | 32789 (47.0%) | 16258 (45.3%) | |
| Drink | No | 37059 (53.1%) | 20062 (55.8%) | <0.001 |
| | Yes | 32748 (46.9%) | 15896 (44.2%) | |
| Chronic disease | No | 28918 (41.4%) | 25064 (44.6%) | <0.001 |
| | Yes | 40889 (58.6%) | 31159 (55.4%) | |

Note: *SHARE – Survey of Health, Ageing and Retirement in Europe.
†Number (percentage [%]) was used to describe categorical variables and the Pearson chi-square test for categorical variables.

## REFERENCES


1 Grimard F, Laszlo S, Lim W. Health, aging and childhood socio-economic conditions in Mexico. J Health Econ. 2010;29:630–40.

2 Wong R, Michaels-Obregon A, Palloni A. Cohort Profile: The Mexican Health and Aging Study (MHAS). Int J Epidemiol. 2017;46:e2.

3 Wong R, Ofstedal MB, Yount K, Agree EM. Unhealthy lifestyles among older adults: exploring transitions in Mexico and the US. Eur J Ageing. 2008;5:311–26.

4 González-González C, Samper-Ternent R, Wong R, Palloni A. Mortality inequality among older adults in Mexico: the combined role of infectious and chronic disease. Rev Panam Salud Publica. 2014;35:89–95.

5 Wong R, Michaels-Obregón A, Palloni A, Gutiérrez-Robledo LM, González-González C, López-Ortega M, et al. Progression of aging in Mexico: the Mexican Health and Aging Study (MHAS) 2012. Salud Publica Mex. 2015;57 Suppl 1:S79–89.

6 Daskalopoulou C, Koukounari A, Wu YT, Terrera GM, Caballero FF, de la Fuente J, et al. Healthy ageing trajectories and lifestyle behaviour: the Mexican Health and Aging Study. Sci Rep. 2019;9:11041.

7 Steptoe A, Breeze E, Banks J, Nazroo J. Cohort profile: the English longitudinal study of ageing. Int J Epidemiol. 2013;42:1640–8.

8 Mindell J, Biddulph JP, Hirani V, Stamatakis E, Craig R, Nunn S, et al. Cohort profile: the health survey for England. Int J Epidemiol. 2012;41:1585–93.

9 Cheshire H, Ofstedal MB, Scholes S, Schroeder M. A comparison of response rates in the English Longitudinal Study of Ageing and the Health and Retirement Study. Longit Life Course Stud. 2011;2:127–44.
10 Mak HW, Noguchi T, Bone JK, Wels J, Gao Q, Kondo K, et al. Hobby engagement and mental wellbeing among people aged 65 years and older in 16 countries. Nat Med. 2023;29:2233–40.
11 Chen X, Smith J, Strauss J, Wang Y, Zhao Y. China Health and Retirement Longitudinal Study (CHARLS). In: Pachana NA, editor. Encyclopedia of Geropsychology. Singapore: Springer Singapore; 2015. p. 1–8.
12 Zhao Y, Hu Y, Smith JP, Strauss J, Yang G. Cohort profile: the China Health and Retirement Longitudinal Study (CHARLS). Int J Epidemiol. 2014;43:61–8.
13 Gong J, Wang G, Wang Y, Chen X, Chen Y, Meng Q, et al. Nowcasting and forecasting the care needs of the older population in China: analysis of data from the China Health and Retirement Longitudinal Study (CHARLS). Lancet Public Health. 2022;7:e1005–e13.
14 Sonnega A, Faul JD, Ofstedal MB, Langa KM, Phillips JW, Weir DR. Cohort Profile: the Health and Retirement Study (HRS). Int J Epidemiol. 2014;43:576–85.
15 Ofstedal MB, Weir DR. Recruitment and retention of minority participants in the health and retirement study. Gerontologist. 2011;51 Suppl 1:S8–20.
16 Fisher GG, Ryan LH. Overview of the Health and Retirement Study and Introduction to the Special Issue. Work, Aging and Retirement. 2018;4:1–9.
17 Börsch-Supan A, Brandt M, Hunkler C, Kneip T, Korbmacher J, Malter F, et al. Data Resource Profile: the Survey of Health, Ageing and Retirement in Europe (SHARE). Int J Epidemiol. 2013;42:992–1001.
18 Börsch-Supan A, Hank K, Jürges H. A new comprehensive and international view on ageing: introducing the 'Survey of Health, Ageing and Retirement in Europe'. European Journal of Ageing. 2005;2:245–53.
19 Yuan Y, Si H, Shi Z, Wang Y, Xia Y, Guan X, et al. Association of Cognitive Frailty With Subsequent All-Cause Mortality Among Middle-Aged and Older Adults in 17 Countries. Am J Geriatr Psychiatry. 2025;33:178–91.
20 Lim JP, Low KYH, Lin NJJ, Lim CZQ, Ong SWX, Tan WYT, et al. Predictors for development of critical illness amongst older adults with COVID-19: Beyond age to age-associated factors. Arch Gerontol Geriatr. 2021;94:104331.
21 Hou C, Ma Y, Yang X, Tao L, Zheng D, Liu X, et al. Disability Transitions and Health Expectancies among Elderly People Aged 65

Years and Over in China: A Nationwide Longitudinal Study. Aging Dis. 2019;10:1246–57.
22 Hardy SE, Dubin JA, Holford TR, Gill TM. Transitions between States of Disability and Independence among Older Persons. American Journal of Epidemiology. 2005;161:575–84.
23 Wang D, Dai X, Mishra SR, Lim CCW, Carrillo-Larco RM, Gakidou E, et al. Association between socioeconomic status and health behaviour change before and after non-communicable disease diagnoses: a multicohort study. Lancet Public Health. 2022;7:e670–e82.
24 Lu X, Yao Y, Jin Y. Digital exclusion and functional dependence in older people: Findings from five longitudinal cohort studies. EClinicalMedicine. 2022;54:101708.
25 Kim J-H, Park E-C. Impact of socioeconomic status and subjective social class on overall and health-related quality of life. BMC Public Health. 2015;15:783.
26 Hu Y, Peng W, Ren R, Wang Y, Wang G. Sarcopenia and mild cognitive impairment among elderly adults: The first longitudinal evidence from CHARLS. J Cachexia Sarcopenia Muscle. 2022;13:2944–52.
27 Song Y, Liu H, Gu K, Liu Y. Association between nighttime sleep duration, nap time, and mild cognitive impairment in Chinese older adults: a cross-sectional study. BMC Public Health. 2024;24:2381.
28 Alimujiang A, Wiensch A, Boss J, Fleischer NL, Mondul AM, McLean K, et al. Association Between Life Purpose and Mortality Among US Adults Older Than 50 Years. JAMA Netw Open. 2019;2:e194270.
